# Attitudes and decision-making about early-infant versus early-adolescent male circumcision: Demand-side insights for sustainable HIV prevention strategies in Zambia and Zimbabwe

**DOI:** 10.1371/journal.pone.0181411

**Published:** 2017-07-27

**Authors:** Sema K. Sgaier, Sunny Sharma, Maria Eletskaya, Ram Prasad, Owen Mugurungi, Bushimbwa Tambatamba, Getrude Ncube, Sinokuthemba Xaba, Alice Nanga, Sehlulekile Gumede-Moyo, Steve Kretschmer

**Affiliations:** 1 Surgo Foundation, Washington, District of Columbia, United States of America; 2 Department of Global Health and Population, Harvard T. H. Chan School of Public Health, Boston, Massachusetts, United States of America; 3 Department of Global Health, University of Washington, Seattle, Washington, United States of America; 4 Ipsos Healthcare, London, United Kingdom; 5 Final Mile Consulting, Mumbai, India; 6 Ministry of Health and Child Care, Harare, Zimbabwe; 7 Ministry of Community Development, Mother and Child Health, Lusaka, Zambia; Cincinnati Children's Hospital Medical Center, UNITED STATES

## Abstract

As countries approach their scale-up targets for the voluntary medical male circumcision program for HIV prevention, they are strategizing and planning for the sustainability phase to follow. Global guidance recommends circumcising adolescent (below 14 years) and/or early infant boys (aged 0–60 days), and countries need to consider several factors before prioritizing a cohort for their sustainability phase. We provide community and healthcare provider-side insights on attitudes and decision-making process as a key input for this strategic decision in Zambia and Zimbabwe. We studied expectant parents, parents of infant boys (aged 0–60 days), family members and neo-natal and ante-natal healthcare providers in Zambia and Zimbabwe. Our integrated methodology consisted of in-depth qualitative and quantitative one-on-one interviews, and a simulated-decision-making game, to uncover attitudes towards, and the decision-making process for, early adolescent or early infant medical circumcision (EAMC or EIMC). In both countries, parents viewed early infancy and early adolescence as equally ideal ages for circumcision (38% EIMC vs. 37% EAMC in Zambia; 24% vs. 27% in Zimbabwe). If offered for free, about half of Zambian parents and almost 2 in 5 Zimbabwean parents indicated they would likely circumcise their infant boy; however, half of parents in each country perceived that the community would not accept EIMC. Nurses believed their facilities currently could not absorb EIMC services and that they would have limited ability to influence fathers, who were seen as having the primary decision-making authority. Our analysis suggests that EAMC is more accepted by the community than EIMC and is the path of least resistance for the sustainability phase of VMMC. However, parents or community members do not reject EIMC. Should countries choose to prioritize this cohort for their sustainability phase, a number of barriers around information, decision-making by parents, and supply side will need to be addressed.

## Introduction

Sustaining the impact of scaled health interventions is a key challenge for the development sector. Few programs take into account sustainability considerations at the early stages of scale-up [1]. Voluntary medical male circumcision (VMMC) is a critical intervention to prevent the spread of HIV in settings with high HIV incidence and low circumcision prevalence [2–5]. In 2011, the World Health Organization (WHO) and the Joint United Nations Programme on HIV/AIDS (UNAIDS) provided a roadmap for scale-up and sustainability through the Joint Strategic Action Framework for VMMC [6]. This roadmap highlighted a two-phased approach. First, a “catch-up” phase where sexually active males would be prioritized to enable near-term reduction in HIV incidence. A global target was set of 20 million circumcisions by 2016 among males aged 15–49. Second, a “sustainability” phase aims to move towards a routine offer of service provision to younger males–infants aged 0–60 days (early infant circumcision–EIMC) and/or adolescents below 14 years old (early adolescent male circumcision–EAMC). The second phase aims to maintain the long-term effects of circumcision on HIV. Fourteen countries in eastern and southern Africa are scaling up VMMC, and 11.7 million circumcisions (over 50% of the “catch-up” target) were performed through 2015 [7].

As countries focus their efforts to achieve their VMMC scale-up targets, early planning for the sustainability phase will ensure a smooth and efficient transition and sustained impact [8]. A key consideration in the design of the sustainability phase is the target population. The Joint Strategic Action Framework provides the possibility of both EIMC and EAMC. Choosing the right target group requires taking into account several factors including the cost of the procedure, feasibility of provision as a routine service, and acceptability (and especially the decision-making process) by the community.

In priority countries 35%-74% of the males being circumcised as part of the “catch-up” phase are adolescents (10–14 years of age), while they represent only 34–55% of the target population to be circumcised, suggesting a high degree of acceptability by circumcising adolescents [9]. Service provision for EIMC is nascent in the context of HIV prevention programming, consisting mostly of experimental pilots, but in many parts of Western and Central Africa, EIMC is routinely offered [10–12]. However, a few studies have provided evidence on the acceptability of EIMC. In a South African study, 67% of fathers and 66% of mothers indicated a willingness to circumcise their babies before the age of 6 weeks [13]. A study in Zimbabwe indicated that 58% of fathers and 60% of mothers demonstrated a willingness to circumcise their newborn sons [14], and another study in Botswana found that 90% of mothers reported they would circumcise their newborn sons, providing it was free of charge, performed in a clinical setting and by a trained healthcare worker [13]. In a study conducted in Zambia, 1,000 mothers of newborn boys across two public clinics were interviewed; 97% of the sample said they probably or definitely would circumcise their newborn son, but only 11% of the sample brought their son back to the clinic to be circumcised [15]. This study concluded that hypothetical acceptability studies in southern Africa imply strong support for EIMC but may not necessarily translate into high uptake [15]. While there is evidence for acceptability of both EIMC and EAMC, a deeper understanding of the decision-making process is needed, especially as it relates to drivers, facilitators, and mental models that guide the decision. Studies to date have not directly asked parents of newborn children to compare EIMC with EAMC, and additional insights into decision-making may lie in this comparison.

The aim of the present study was to use an integrated methodology to explore the comparative preference and decision-making path for EIMC versus EAMC by parents, caregivers and healthcare providers. In particular, we sought to understand the difference in decision-making processes of parents when choosing whether to circumcise their baby boy compared with their adolescent boy. The study was conducted in Zambia and Zimbabwe to inform their national sustainability strategies and implementation plans regarding demand-side preferences and beliefs, as they also consider other important factors such as the feasibility, scale-up and cost-effectiveness of each. [16, 17].

## Methods

### Instrumentation and data collection

Prior to conducting the study, it was reviewed and approved by the Medical Research Council of Zimbabwe institutional review board in Zimbabwe, the Research Council of Zimbabwe and the ERES Converge institutional review board in Zambia. Primary data was collected in three phases through semi-structured qualitative interviews and structured quantitative surveys. Each phase informed the design of the subsequent phase in order to give a holistic analysis framework and provide robust evidence. Phases 1 and 2 were analyzed in full prior to the creation of phase 3 study materials.

For each phase of the research, written consent was obtained from respondents. For illiterate respondents, agreement to participate in the study as illustrated by an inked thumb print were treated as written consent. Interviewers used a consent form that they read and explained to potential study participants. Participants were informed that they could terminate the interview at any point in time without any consequences. A document summarizing respondents’ rights was provided, including the telephone number and contact information for the principal investigator (PI). This one page document was translated into local languages. The IRBs in each country approved this consent procedure and the relevant forms and documents in English and local languages.

Phase 1 consisted of qualitative in-depth interviews conducted with new and expectant fathers, mothers, paternal grandfathers and paternal grandmothers of baby boys younger than 2 months, given their crucial role as influencers of family decisions [18]. The interviews were conducted with 18 mothers and fathers each and of 9 grandmothers and grandfathers each in each country. Furthermore, antenatal and neonatal healthcare workers were interviewed (n = 18 per country) to identify their interest and role in promoting sustainable circumcision. This phase used a semi-structured, open-ended qualitative discussion guide to record a wide range of thoughts and attitudes and inform the designs for Phases 2 and 3.

Phase 2 consisted of a qualitative behavioral economics assessment (Ethnolab) to understand context, mental models, and emotions that influence parents’ decision-making in relation to circumcising their infant boys (younger than 2 months). Ethnolab is a game-based research technique designed to minimize the impact of biases in decision-making. In research settings, biases such as fear of value judgment, social desirability, and expectation of higher self-control in future make respondents claim behaviors that may not play out in real life, i.e., “Say–Do” gaps.

In Ethnolab, 10 participants play the game at a time. The profile of all participants is similar in terms of their income, age, and gender. Participants are exposed to unique situations–a narrative with audio-visual aid or through a live narration. The narrative ends with a decision conundrum, exposing a set of 3–4 different scenarios among which the participants must choose. Participants make their choice based on what they believe the majority in the group will select as the most likely outcome among the options. If the response of the participant matches that of the majority response in the group of 10 people playing the game, the participant stands to win cash (in Zambia, 2 Kwacha for the right answerer, with a maximum of 24 Kwacha to be made; in Zimbabwe, $1 for the right answer, with a maximum of $12 to be made). The game generates responses which are representative of the participants’ emotions, mental models, and biases, rather than their deliberative and rational analyses regarding why they ‘think’ they behave. This approach addresses both individual and social biases, such as fear of value judgment and social desirability. Participants are asked to play the game over 12 rounds. Following the game, participants are engaged in a short conversation regarding their experience, decisions, and preferences in the game.

Hypotheses on behavioral outcomes to be tested were developed based on our review of the available literature and the findings from Phase 1 qualitative research (see Table 1). These hypotheses were used to construct a number of decision scenarios which parents could face in the context of circumcising their boy. The Ethnolab was used primarily to validate or negate these hypotheses.

**Table 1:** Hypotheses tested in the Ethnolab.

Observation	Hypotheses
Timing of circumcision. Fathers and mothers have different preferences as far as life stage of circumcision is concerned	1. Mothers prefer soon after birth as the child will feel least pain then 2. Fathers worried about responsibility attribution and want the child to grow up and take his own decision 3. Fathers believe boys will feel less pain that infants 4. Fathers believe the best time for circumcision is when boys are about to become sexually active
Time of advocating to parents to circumcise their male child can make a difference to the decision	1. On confirmation of pregnancy 2. 2 months before delivery 3. 2 days before delivery 4. Soon after delivering the child
Anticipated emotions can impact decisions.	Salient emotions may be varied and include: 1. satisfaction 2. pride 3. relief
Tapping the right motivation among fathers and mothers is key to driving the decision about circumcision	1. Here-and-now benefits of hygiene as boys don’t bathe regularly 2. Boys may not be able to tolerate the pain of being circumcised once they grow up 3. Cannot leave an important decision like this to the boy
How others perceive a mother who has circumcised her boy has a bearing on the decision	1. Progressive 2. nurturing 3. aware 4. one who gets easily influenced by others
Barriers to decision-making are varied	1. Cultural norms 2. Fear of failed surgery 3. Social proof–not enough circumcised kids 4. Temporal factors–benefits are in the future, so why circumcise now? 5. Mothers advocating circumcision may be seen as promiscuous or as disrespecting grandparents 6. Nature of diseases will change in future 7. Son will grow up and blame us for taking such an important decision ourselves 8. Son will grow up to be sexually responsible
Role of mothers in decision-making needs to be established	1. Women lack the confidence to take a decision so leave it to the Husband 2. Lack of self-belief 3. Anticipation of guilt–what if something goes wrong?
Grandparents are giving way to other influencers	1. Father’s decision is all that matters 2. The nurse helping during delivery can be a strong influence on the father 3. Health worker/tribal leader can be an effective influencer
Mental models around grandparents are changing	1. A belief that grandparents are outdated in their thinking 2. Health issues have changed so much that grandparents’ advice has little value 3. Circumcision will be a norm by the time the boys grow up
Grandparents and fathers have higher influence in decision-making	1. Self-image is a key driver for fathers–a father who is circumcised himself is more likely to get his son circumcised 2. Mothers who believe that circumcision would lead to health and hygiene can take the decision 3. It’s the grandparents who have a significant say as it’s part of the tradition

In total, 48 expectant or new mothers (6 groups of 8 respondents each) and 48 expectant or new fathers (also 6 groups of 8 respondents each) of newborn baby boys younger than 2 months participated in the Ethnolab in each country. Districts (provinces) where the study was conducted were Kitwe (Copperbelt), Katete (Eastern), and Lusaka (Lusaka) in Zambia, and Harare (Harare) Bulawayo (Bulawayo) and Kwekwe (Midlands) in Zimbabwe.

Phase 3 of the research was a quantitative survey that used a structured questionnaire, designed to test with a larger, nationally representative sample the insights and hypotheses generated from the qualitative phases. This phase was focused on EIMC, given that there are fewer data on infant circumcision and less experience with it. The Phase 3 research explored general attitudes towards health outcomes of infant boys, knowledge of HIV, general knowledge of male circumcision, knowledge of EIMC, attitudes towards EIMC, preferred location for EIMC services and willingness to pay for services. New and expectant mothers (n = 500 per country) and fathers (n = 500 per country) were interviewed face-to-face. Sample sizes of 500 expectant mothers and fathers each were determined based on the expectation to test nested cross-tabulations for significant differences with a minimum sample of n = 30 to 50 in the smallest respondent filter to be tested. In collaboration with the ministries, 8 (from 10) provinces, deemed representative of the country’s non-circumcising community, were selected in Zambia, and all 10 provinces in Zimbabwe. Within each province, interviews were conducted in urban, peri-urban and rural areas. The sample in each province mirrored the VMMC target per region (80% coverage at district level) set by the national VMMC program in each country. Households were randomly sampled in the selected provinces and a mothers or fathers were identified and approached in each household for the interview. If more than one eligible respondent lived in the household, selection among them was made by random selection using a table of random numbers. If a father lived in the household but were not currently present, the interviewer arranged to return to the household to interview that father at a later time. Interviews were conducted in private settings identified within or close to the home. All responses were directly entered into the survey, programmed on a mobile device, by the interviewer.

### Analysis

Qualitative interviews in phase 1 were transcribed in English. Responses to questions were grouped together by content to pick out dominant themes as well as differences in opinions to test during the Ethnolab (Phase 2) and quantitative survey (Phase 3). Transcripts from fathers, mothers, grandfathers, grandmothers, and healthcare workers were all analyzed as separate groups. Findings were synthesized by group and compared with those from other groups.

Quantitative data from Phase 3 were analyzed using the SPSS statistical package (version 6.0.1). Data for mothers and fathers were analyzed separately, and statistical significance comparisons were made at the question level, including across provinces, between HIV status (positive or negative), between spontaneous awareness of VMMC or not, and between circumcision status of the father (circumcised or not).

## Results

### General knowledge about EIMC

The Phase 3 quantitative survey provided insights into knowledge of EIMC and sources of this knowledge. Among the respondents from Zambia, 34% had heard about infant circumcision, compared with 67% in Zimbabwe. The most common source of information on EIMC in both countries was from healthcare providers (41% in Zambia and 58% in Zimbabwe), with this being a more likely source for expectant mothers than for expectant fathers (Table 2). The second most common sources of information in both countries were the same-sex friend and spouse. Expectant fathers were more likely to receive information on EIMC from their spouses than vice versa (34% of expectant fathers vs. 15% of expectant mothers in Zimbabwe and 10% of expectant fathers vs. 4% of expectant mothers in Zambia picked this a source of information for EIMC). Other common sources of information in both countries included community mobilizers, television, and radio.

**Table 2:** Sources of information about circumcising baby boys, ages 0 to 2 months after birth.

	Zambia			Zimbabwe		
	TOTAL Who Have Heard of EIMC (n = 362)	Expectant Father (n = 169) D	Expectant Mother (n = 193) E	TOTAL Who Have Heard of EIMC (n = 365)	Expectant Father (n = 163) D	Expectant Mother (n = 202) E
Doctor, nurse, or healthcare worker	41%	35%	46% (D) (p = 0.038)	58%	43%	70% (D) (p = 0)
Female friend	13%	7%	19% (D) (P = 0.0006)	12%	4%	18% (D) (p = 0)
Male friend	12%	20% (E) (p = 0)	4%	12%	21% (E) (p = 0)	4%
Community mobilizer	9%	12%	6%	17%	14%	19%
Television	8%	10%	7%	12%	13%	11%
Other family member	8%	8%	8%	4%	4%	3%
Radio	7%	8%	6%	13%	13%	12%
Your spouse or partner	7%	10% (E) (p = 0.01428)	4%	24%	34% (E) (p = 0)	15%
Your mother	4%	4%	4%	2%	2%	1%
Your brother	4%	5%	3%	4%	5%	3%
Your father	2%	2%	2%	1%	2%	*
Posters/Billboards/Signs	2%	2%	1%	5%	8% (E) (p = 0.01596)	2%
Teacher	1%	2%	1%	1%	-	1%
Church leader or other religious leader	1%	2%	1%	1%	2%	1%
Newspapers or magazines	1%	2%	1%	3%	2%	3%
Your grandfather	1%	2%	-	1%	2%	*
Your grandmother	1%	1%	1%	2%	2%	2%
Chief or other community leader	1%	1%	1%	*	1%	-
Your grandmother	1%	1%	1%	1%	2%	*
Internet	1%	1%	-	4%	4%	3%
Celebrity	*	-	1%	1%	1%	-
Your son	-	-	-	-	-	-
Other–specify	25%	27%	23%	3%	2%	3%
Cannot remember/cannot say	2%	2%	2%	1%	2%	-

D: Statistically significant higher value among mothers compared with fathers.

E: statistically significant higher value among fathers compared with mothers.

* Sample size too small.

### Perceived current age and ideal age for circumcision

**Fig 1:**
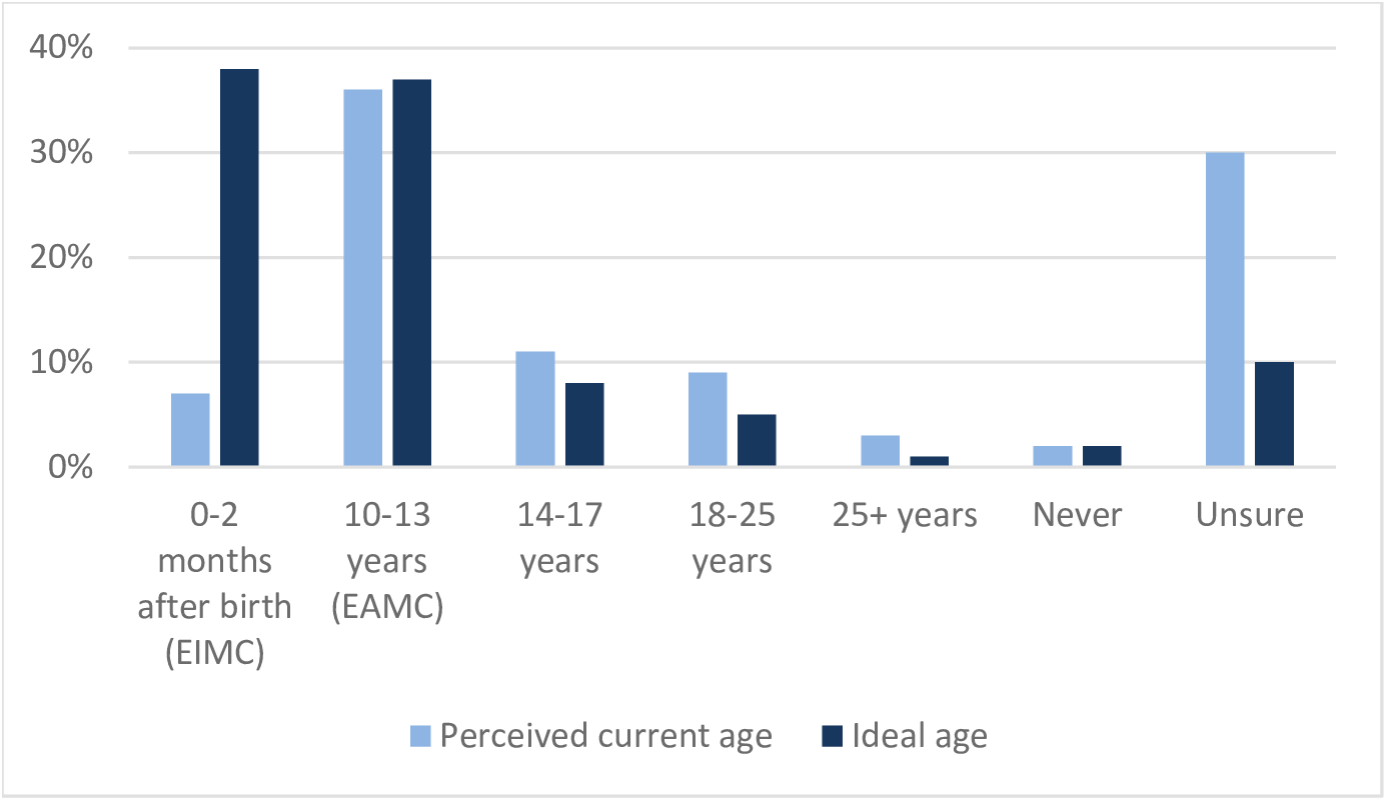
Perceived current age of men receiving MC vs. perceived ideal age for MC uptake, Zambia.

**Fig 2:**
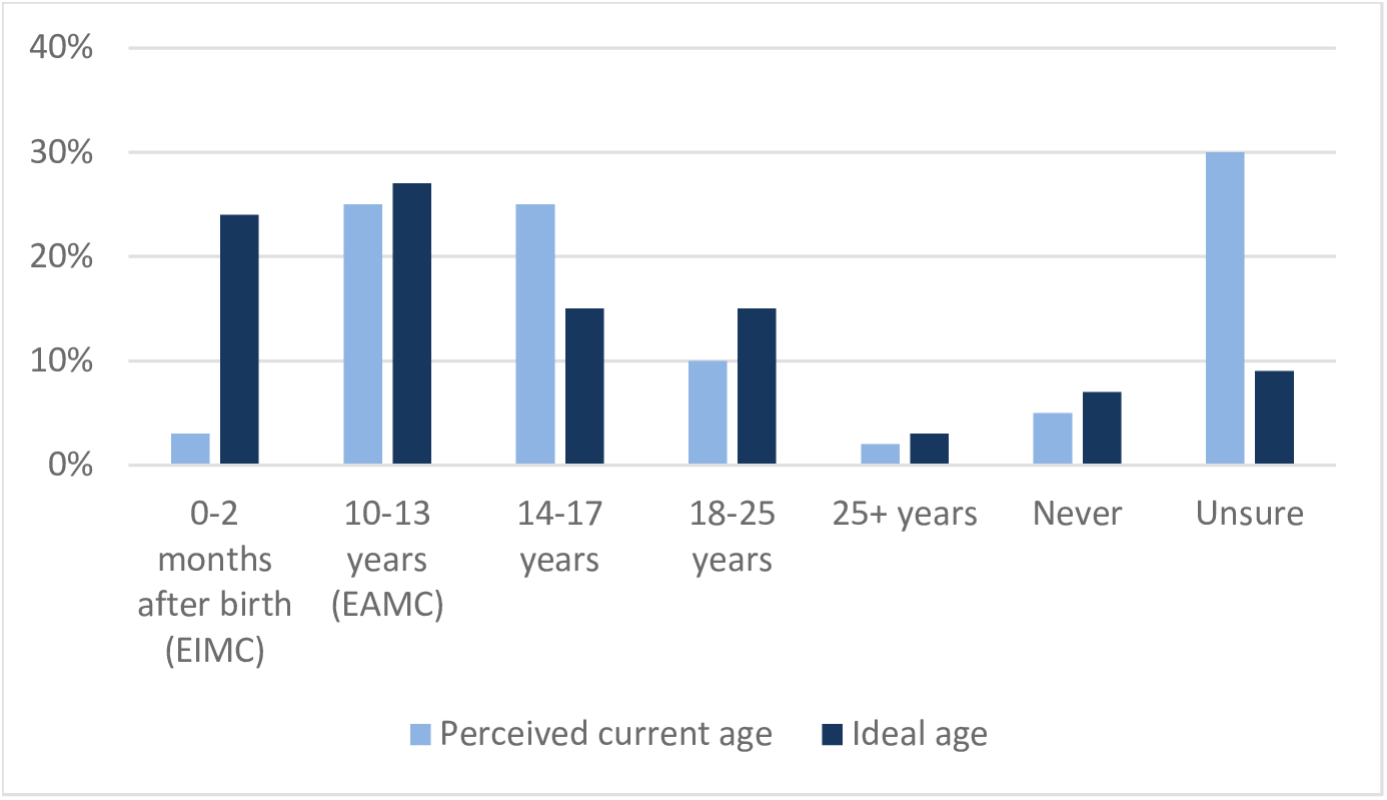
Perceived current age of men receiving MC vs. perceived ideal age for MC uptake, Zimbabwe.

In the Phase 3 quantitative survey we asked mothers and fathers, each, the most common age at which males in their community tend to get circumcised, medically or traditionally, if they do at all, and what they thought is the ideal age for a boy or man to be medically circumcised (Figs 1 and 2). Circumcisions were more widely perceived to happen among adolescent boys of less than 14 years of age in Zambia (36% of respondents) than in Zimbabwe (25% of respondents). In both countries, only a small proportion of respondents believed that infants are circumcised (7% in Zambia, 3% in Zimbabwe). However, the distribution of the ideal age for circumcision shifted to younger males in both countries. In Zambia, three-quarters of respondents felt that circumcising prior to timing of sexual debut (infant to 14 years) is ideal, compared with half in Zimbabwe. The split between viewing infancy or early adolescence as the ideal timing was almost equal in both countries (38% from the quantitative survey viewed infancy as ideal vs. 37% viewing adolescence as ideal in Zambia, 24% vs. 27% in Zimbabwe). No differences were observed between fathers’ and mothers’ answers.

Beliefs about the ability of the circumcision wound to heal faster (42% in Zimbabwe, 28% in Zambia), the boy’s likelihood not to suffer as much pain (27% in Zimbabwe, 23% in Zambia), and other personal benefits (e.g., the boy will not have to make the decision later in life, he will be taken care of by his parents and others, and circumcision is better before being sexually active) were cited as the most common reasons for choosing 0–2 months as the ideal age to circumcise (S1 Table). In Zimbabwe, those who believe adolescent circumcision is best are driven by the boy being old enough to making his own decision (16%), thereby lessening the parents’ responsibility to decide. They also consider the benefits of circumcising before sexual debut (12% in Zimbabwe, 15% in Zambia) and that the wound heals faster (13% in Zimbabwe, 7% in Zambia). While pain was also one of the reasons chosen by these respondents, it was more because they thought the boys were old enough to handle the pain (9% in Zimbabwe, 6% in Zambia) and to some degree would not suffer as much (6% in Zimbabwe, 5% in Zambia). They also preferred EAMC because they believed that this is the most appropriate age for the procedure (13% in Zimbabwe, 23% in Zambia).

### Attitudes towards EIMC

In the Phase 3 interviews, almost half of the parents interviewed perceived that the community would not accept EIMC (49% in Zambia and 43% in Zimbabwe picked 1 or 2 on a 7-point scale, with 1 being not acceptable at all) (Table 3). Among the respondents, 27% in Zambia and 13% in Zimbabwe felt that EIMC was acceptable to the community (those who picked 7 or 6 on a 7-point scale with 7 being completely acceptable).

**Table 3:** Perceived acceptability of EIMC within community.

	Zambia			Zimbabwe		
	Total	Fathers	Mothers	Total	Fathers	Mothers
	**n = 1,005** ***100%***	n = 503 *100%*	n = 502 *100%*	**1,000** ***100%***	**500** ***100%***	**500** ***100%***
Top 2 Ratings (6 or 7)	**266** ***26%***	137 27%	129 26%	**129** ***13%***	70 14%	59 12%
Middle 3 Ratings (3, 4 or 5)	**244** **25%**	121 24%	123 24%	**439** **44%**	216 43%	223 44%
Bottom 2 Ratings (1 or 2)	**495** ***49%***	245 49%	250 50%	**432** ***43%***	214 43%	218 44%

In your opinion, how acceptable to people in your community would be circumcising baby boys at the age of 0 to 2 months old? Please rate your view of the acceptability using the following 7-point scale where 1 means ‘Not At All Acceptable’ and 7 means ‘Completely Acceptable’.

When respondents were asked whether they would circumcise their baby boys if the procedure was offered for free, 54% vs. 42% in Zambia, and 36% vs. 52% in Zimbabwe, said they definitely would or probably would vs. definitely or probably would not (Table 4). In both countries, those respondents who could spontaneously recall VMMC as a method of HIV prevention, knew someone who is HIV positive, or knew someone who had circumcised a baby, and those from higher socioeconomic groups (based on DHS calculator, grouping respondents into socio status by income, wealth and ownership of household goods), were more likely to circumcise their baby boys. Protection against infection (HIV and other sexually transmitted infections) (25% in Zambia and Zimbabwe), cleanliness (14% in Zambia and 18% in Zimbabwe) and feeling less pain (13% in Zambia and 11% in Zimbabwe) were cited as the top benefits of circumcising infants (Table 5). However, over 30% of the respondents in both countries stated that they had not heard of any benefits of EIMC. When asked about concerns, 34% of respondents in Zambia and 26% in Zimbabwe stated that they had no concerns about circumcising infants. For those that did have concerns, the top reasons cited included belief that the infant is too fragile and young, the need for after-care, the quality of the procedure (i.e., something could go wrong or the procedure might not be done properly), the pain may be too much for the child, and it could lead to death. During qualitative interviews, parents had difficulty visualizing the skin being removed from the small penis and hence difficulty in understanding the procedure.

**Table 4:** Likelihood to circumcise baby if service was free.

	Zambia			Zimbabwe		
	Total	Fathers	Mothers	Total	Fathers	Mothers
	**n = 1,005** ***100%***	n = 503 *100%*	n = 502 *100%*	**1,000** ***100%***	500 *100%*	500 *100%*
Top 2 Ratings (6 or 7)	**453** ***45%***	217 43%	236 47%	**364** ***36%***	179 36%	185 37%
Middle 3 Ratings (3, 4 or 5)	**141** **14%**	74 15%	67 13%	**138** **14%**	61 12%	77 15%
Bottom 2 Ratings (1 or 2)	**411** ***41%***	212 42%	199 40%	**498** ***50%***	260 52%	238 48%

How likely would you be to circumcise your baby boy when he is 0 to 2 months old, if the procedure was free of cost? Please answer using the following 7-point scale where 1 means ‘Definitely would not’ and 7 means ‘Definitely would’.

**Table 5:** Main perceived benefits and concerns of circumcising infant boys.

	Zambia			Zimbabwe		
	Total	Fathers	Mothers	Total	Fathers	Mothers
	n = 1,005 *100%*	n = 503 *100%*	n = 502 *100%*	1,000 *100%*	500 *100%*	500 *100%*
**BENEFITS**						
Protection against infection / diseases	249 25%	110 22%	139 28%	252 25%	126 25%	126 25%
Cleanliness	145 14%	86 17%	59 12%	179 18%	89 18%	90 18%
Less Painful	128 13%	72 14%	56 11%	113 11%	55 11%	58 12%
**CONCERNS**						
Pain	115 12%	63 13%	52 10%	175 18%	84 17%	91 18%
Infection / diseases	119 12%	53 11%	66 13%	119 12%	55 11%	64 13%
Quality of Procedure	66 7%	35 7%	31 6%	140 14%	77 15%	63 13%
Child to young / small / fragile / Can do when older	108 11%	65 13%	43 9%	86 9%	46 9%	40 8%
Better he makes his own decision / he could blame me later	8 1%	7 1%	1 <1%	91 9%	50 10%	41 8%
Nothing / no concerns	341 34%	155 31%	186 37%	256 26%	116 23%	140 28%

Net coded responses from open-ended verbatim answers; multiple responses from single respondent possible.

### Preferred location and willingness to pay for services

**Fig 3:**
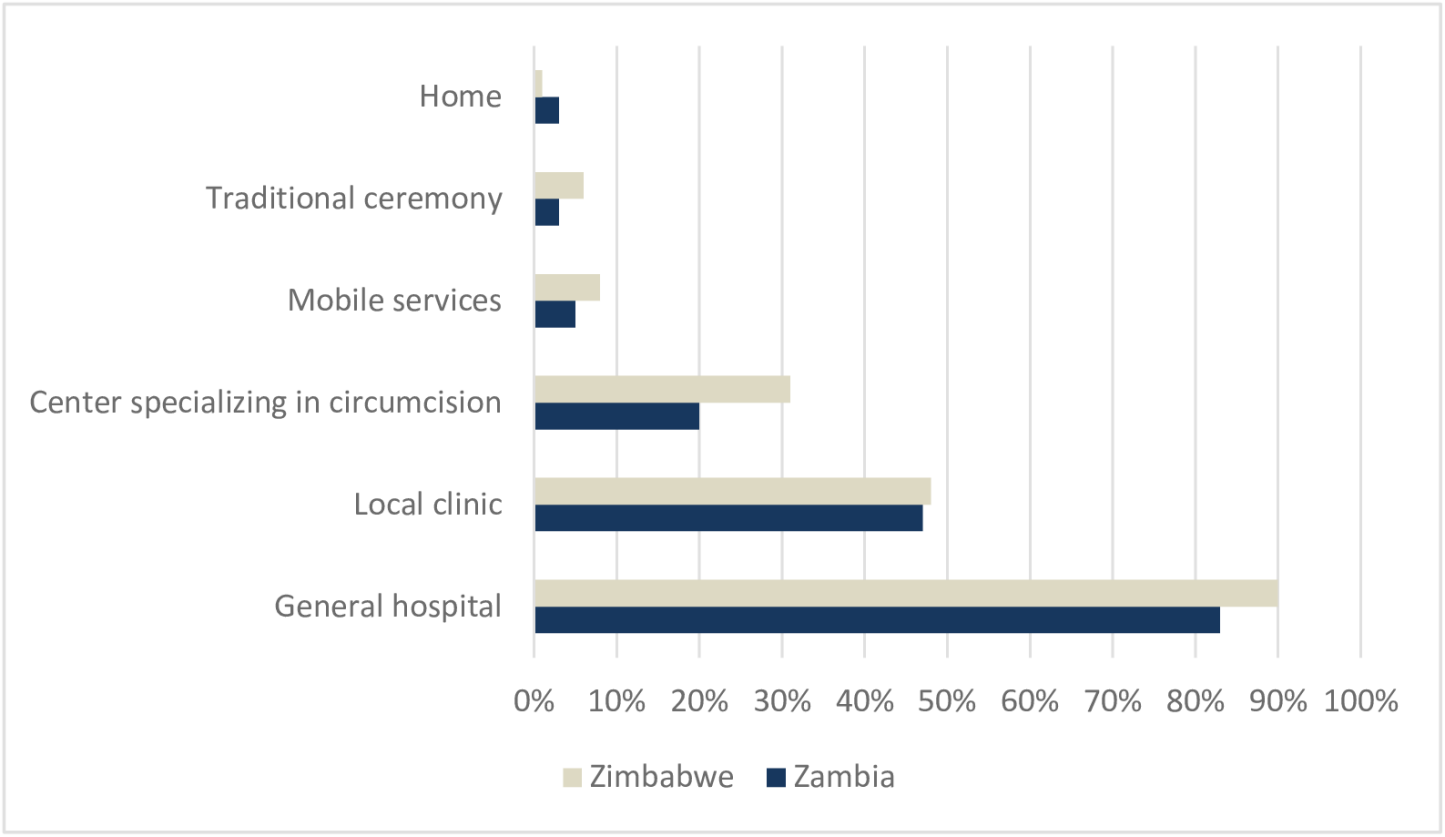
Settings where parents would consider having their infant boy get circumcised.

Parents who demonstrated willingness to circumcise their baby boy would prefer to do so in the general hospital (83% of the respondents in Zambia and 90% in Zimbabwe) (Fig 3). Data from the qualitative interviews suggest that parents believed that the clinic was the best suitable place for EIMC, as complications could be addressed immediately. Close to 50% of respondents in both countries stated that they would consider the local clinic as well. A circumcision clinic was more acceptable in Zimbabwe than in Zambia. Preference for mobile service–circumcisions to be conducted at home by a trained provider, or circumcision through traditional services–was very low. No difference in preferred setting was observed between mothers and fathers.

We asked parents who indicated that they were likely to circumcise their baby boy (by rating their likelihood as 6 = probably or 7 = definitely using a 7-point Likert scale) how likely they would be to follow through if they had to pay a fee for the service (Table 6). In Zambia, close to 80% of parents are willing to pay a small fee (7.5 Zambian kwacha or $1.00 USD). Willingness to pay drops as the fee increases. In Zimbabwe, only a little over 50% of the parents were inclined to pay, even at the most minimal fee of $1.00 USD. Only at the $10 price point were fathers more likely to pay than mothers. Fathers who are circumcised themselves are more willing to accept a cost for EIMC (regardless of the amount), compared with fathers who are not circumcised.

**Table 6:** Parents’ willingness to pay to circumcise their baby boys.

Zambia						Zimbabwe					
	TOTAL	Father	Mother	Father, person-ally circum-cised	Mother, partner circumci-sed		TOTAL	Father	Mother	Father, person-ally circum-cised	Mother, partner circumci-sed
Kwacha (USD)	(N = 620) %	(N = 304) %	(N = 316) %	(N = 139) %	(N = 145) %	USD	(N = 581) %	(N = 287) %	(N = 294) %	(N = 94) %	(N = 103) %
7,5 (1.00)	80	80	79	77	81	1.00	56	55	56	69 ^	71
20 (2.67)	69	71	66	71	71	2.50	53	54	51	68 ^	66
35 (4.67)	59	61	57	65	63	5.00	49	53	46	68 ^	59
55 (7.33)	52	54	50	60	54	7.50	45	49	41	65 ^	56
75 (10.00)	47	49	45	56	50	10.0	43	47 (E)	38	62 ^	54
110 (14.67)	43	47	40	53	46	15.0	41	44	38	55 ^	52

### Attitude of healthcare providers

Qualitative interviews with ANC and PNC healthcare providers included one working in a clinic in Zambia that also provided EIMC, but none providing EIMC services in Zimbabwe. These interviews highlighted that providers were well informed about male circumcision and saw significant benefits to circumcising males in infancy (hygiene, future protection from sexually transmitted infections and HIV, wound’s ability to heal quickly, less loss of blood, and overall less pain experienced). Nurses felt that their facilities would need equipment, space, and trained staff to be able to perform EIMC services (as also would be true to provide EAMC services). Almost none of the nurses felt that they could currently absorb EIMC services. They felt that there was currently limited opportunity to sensitize parents on EIMC and that the mother’s knowledge of health interventions did not necessarily transfer into the household, where a lot of the decisions were made jointly with, or exclusively by, other family members.

### Revealed decision-making process

While the Phase 1 qualitative and Phase 3 quantitative surveys uncovered stated attitudes towards EIMC and EAMC, we used the Ethnolab to uncover underlying drivers of decision-making by parents of infant boys. When presented with competing scenario choices for EIMC vs. EAMC, both mothers and fathers preferred circumcising their boys at adolescence. The reason for this was the desire among parents to ‘abdicate responsibility’ for the choice. The choice would be entirely with the parents for EIMC, whereas with EAMC the boy himself brings the idea of circumcision to the family home from school.

The anticipated regret for an action which goes wrong, such as an adverse event in a circumcision procedure, was greater than regret for not taking an action. The uncertainty parents had about the procedure with infants led to exaggerated claims about dangers and the vulnerability of the infant. Familiarity with what EIMC procedure involves was quite low for both countries, and relatively lower in Zambia.

The sense of vulnerability of the child and anticipation of regret was higher for EIMC than for EAMC. The fear of pain for the child was also higher for EIMC. Both of these findings were confirmed by Phase 3 survey results. Fathers believed that adolescents could tolerate the pain. The emotions of shame and pain felt by adolescents are much better managed in EAMC than in adult MC, thanks to the group dynamics of sending boys to be circumcised with their friends or classmates. Here-and-now benefits scored high, which explains the preference for EAMC. Benefits of EIMC seemed too distant in the future for it to appear relevant.

### Influencers and decision-makers of EIMC

Close to 70% of the parents in both countries stated that the father was the most important decision-maker for circumcising an infant, compared with 27% who believed it to be the mother. This was also confirmed in the Ethnolab, where it was clearly the father’s decision from the perspective of all participants. Fathers who were circumcised themselves were more likely to circumcise their sons. In the Ethnolab, we also found that women had better awareness than men, but that mothers lacked confidence in making the decision and abdicated it to their husbands. In Zimbabwe, parents placed greater weight on themselves, compared with their partners, in influencing decision-making. Healthcare providers played a minor role (10% of respondents in Zambia and 5% in Zimbabwe chose healthcare providers as the most important person in deciding whether infant would get circumcised). When asked who else would influence decision-making, if the father was not mentioned as the primary decision-maker, mothers were prominent (58% in Zambia and 70% in Zimbabwe), followed by the baby’s father (25% in Zambia, 27% in Zimbabwe), the paternal grandfather (21% in Zambia, 14% in Zimbabwe) the paternal grandmother (16% in Zambia, 14% in Zimbabwe) and then the maternal grandfather or grandmother (11% and 10% in Zambia, and 8% and 7% in Zimbabwe). The Ethnolab uncovered that grandparents had a small influence and their influence seemed to be diminishing, especially since there was a strong belief that circumcision will become the norm by the time the boys grow up.

**Fig 4:**
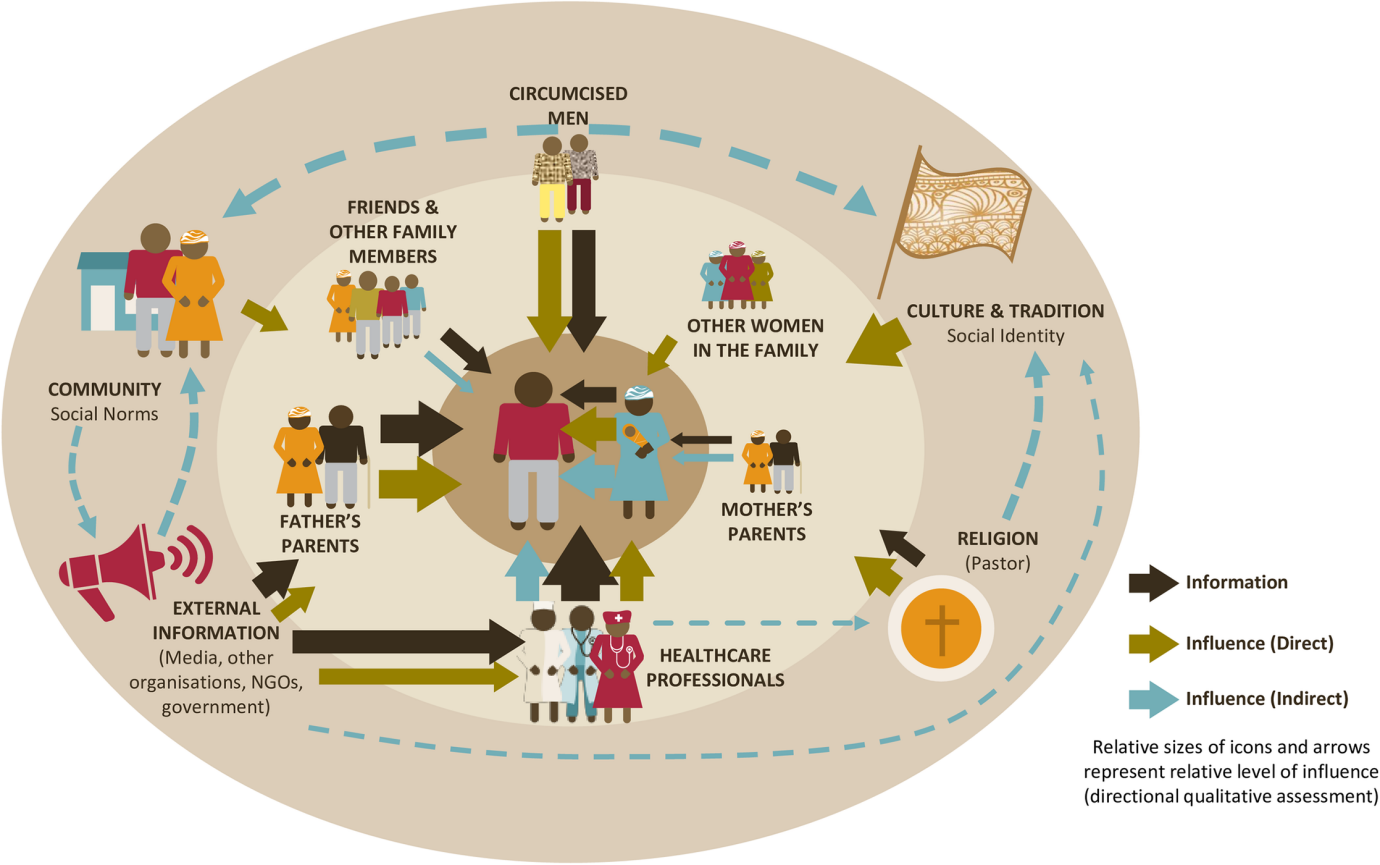
The influencer sphere–complex set of influences for parents’ decision-making.

Our analysis uncovered the complex network of direct and indirect influences on EIMC in the community (Fig 4). Both parents were affected by what their own parents thought (Table 7). A high proportion of fathers and mothers would not have their child circumcised if their spouse or their parents did not agree. This was especially the case for mothers in Zimbabwe, where 81% of mothers stated that they would not circumcise their infant boys if their husband did not agree, and 71% would not do so if their husbands’ parents did not agree. Healthcare providers were a very important source of information, but were less influential on the decision-making process. Religious beliefs and social norms influenced the decision-making, though more so in Zambia than in Zimbabwe.

**Table 7:** Influences of parents in decision-making to circumcise baby boy.

	Zambia		Zimbabwe	
	Mothers	Fathers	Mothers	Fathers
% would NOT circumcise boy if…				
Spouse against it	55%	43%	81%	69%
Own parents against it	42%	39%	58%	52%
Spouse’s parents against it	48%	40%	71%	57%
**% WOULD circumcise boy because it’s becoming a social norm**	48%	45%	26%	28%
**% for whom their religious beliefs would strongly influence their decision**	34%	33%	26%	19%

## Discussion

This study provides community and healthcare provider-side insights to help policy-makers in Zambia and Zimbabwe strategize and design programs to sustain the impact of the current VMMC services (as part of the “catch-up” phase), especially relating to the choice of the appropriate age cohort to prioritize. Our findings are in line with much of what has been uncovered on EIMC acceptability by previous research studies, but also provide new insights [19]. To the best of our knowledge, this is the first study that directly compares the attitudes towards EIMC and EAMC of parents of infants, as well as their decision-making processes for these procedures at the time of their boys’ infancy.

It should be stated here, that while our study sought to compare attitudes regarding EIMC versus EAMC among parents and providers, a key limitation of the study was that our research was conducted only with expectant or recent parents of infants and providers of antenatal and post-natal care services. The results are thus representative of the views of decision-makers and providers for infant boys, and do not reflect the same for adolescent boys. Since the consent decisions by parents to circumcise at infancy versus at adolescence are essentially two separate sequential decisions–yes at infancy or delay decision to later, then yes or no at adolescence–the results of our study should be viewed as reflecting understanding of the first decision (at infancy) and not of the decision-making parents make when their boys are adolescents.

Our results suggest differences in attitudes towards EAMC vs. for EIMC among parents of infant boys, and in the decision-making that parents undergo for these two procedures. EAMC is more accepted and is the path of least resistance compared with EIMC, possibly because it already occurs as part of the “catch-up” phase. Close to 50% of the circumcisions in both countries today take place among early adolescent males, illustrating parents’ willingness to consent to their boys being circumcised at this age. Furthermore, the decision-making burden for EAMC is perceived as shared with the boy and his parents. There is large degree of uncertainty regarding the EIMC procedure, likely not in part due to the general lack of service provision for EIMC in each country. When asked to hypothetically consider EIMC for their infant boys, some parents feel that the baby is ‘too small’ and they cannot visualize how much skin will be removed. Sexual hygiene and the protection against HIV and sexually transmitted infections that programs prioritize today to young adolescents are perceived as closer to near-term benefits for these boys, as compared with for infants, which makes them more relatable to parents for EAMC decisions.

Despite the relative success of EAMC to date, and the propensity of parents to favor EAMC, EIMC still can offer a compelling solution. Benefits that parents see with circumcising infants include faster healing, less pain experienced, and that the child will not remember the pain. However, EIMC faces strong competition from EAMC, and several community and healthcare-side challenges would need to be overcome for EIMC to be a scalable intervention for the sustainability phase. Almost half of the parents interviewed perceive that their community would not accept EIMC. Between 40–50% of the parents said they would not circumcise their infants even if services were offered for free. At the community level, the challenges include conveying information about the benefits and process of EIMC to fathers, managing the uncertainties associated with the procedure, and addressing the emotional impact on parents in their decision-making process. Parents feel quite isolated when making the decision to circumcise their baby boy as compared with their adolescent son. For EAMC, parents feel some of the burden of responsibility is shared with the boy himself, removing potential blame. The benefits of circumcising before sexual debut are also well understood and salient.

Whilst antenatal nurses believe that EIMC is an effective circumcision solution, there are supply-side factors to address, such as training, space, time, and protocol. Furthermore, parents feel that the general hospital is the ideal setting to circumcise their baby boy. Should something go wrong, they trust that the hospital is the most appropriate place for rapid expert attention.

There are a few strategies that countries can prioritize, should they wish to scale up EIMC services. Males who are circumcised during the current ‘catch-up’ phase could be sensitized on EIMC to help create a culture of normalcy. Programs could also consider charging a small fee for the services, as parent are willing to pay and this would relieve the cost-side burden of scaling an indefinite national health program. Healthcare providers are one of the most trusted sources of health information; however, they lack many opportunities to sensitize new parents and have a weak link to the father, who is the main decision-maker. Because of their bias towards the present, parents tend to excessively discount long-term benefits, so the here-and-now benefits of EIMC should be highlighted. This is further necessitated by parents’ perception that although HIV risk is real, the probability of their son contracting the infection is uncertain. This uncertainty barrier would need to be addressed. Uncertainty leads parents to indecision–a state where they believe in the benefits but do not understand the risks and therefore exaggerate them to the point of not being able to decide. Knowledge barriers around the process should be relatively simple to correct, and this would be effective in reducing the over-exaggeration of potential risks of EIMC. Novel approaches are needed to either sensitize couples together, prior to or early on in their pregnancy, to give them enough time to decide, and to directly reach the fathers. Fathers seem to be especially influenced by their parents. Therefore, programs should explore creating direct channels of communication between healthcare providers, the father, and his parents. Broader community-level sensitization is also needed, given that cultural norms and religious factors do play a role in decision-making. Scalable approaches can include media campaigns and direct advocacy with community leaders and gatekeepers.

## Supporting information

S1 Table Reasons for choosing EIMC or EAMC as ideal age for circumcising males. (DOCX)

S1 File Phase I discussion guide-ANC-PNC providers. (PDF)

S2 File Phase I discussion guide-EIMC provider. (PDF)

S3 File Phase I discussion guide-grandparents. (PDF)

S4 File Phase I discussion guide-parents. (PDF)

S5 File Phase II ethnolabs scenarios. (PDF)

S6 File Phase III quant questionnaire. (PDF)
