# Supplementary material for: Attitudes and decision-making about early-infant versus early-adolescent male circumcision: Demand-side insights for sustainable HIV prevention strategies in Zambia and Zimbabwe
Source: PLoS One. 2017 Jul 27;12(7):e0181411. doi: 10.1371/journal.pone.0181411 (PMC5531536; doi:10.1371/journal.pone.0181411)
Supplement: S1 Table — (DOCX) [file pone.0181411.s001.docx]

**Supplementary Table 1: Reasons for choosing EIMC or EAMC as ideal age for circumcising males**

|  | **0-2 months old** | | | | | |  | **10-13 yrs. old** | | | | | |
| --- | --- | --- | --- | --- | --- | --- | --- | --- | --- | --- | --- | --- | --- |
|  | **Zimbabwe** | | | **Zambia** | | |  | **Zimbabwe** | | | **Zambia** | | |
| **Reason** | **Father (n=117)** | **Mother (n=124)** | **Total (n=241)** | **Father (n=180)** | **Mother (n=195)** | **Total (n=275)** |  | **Father (n=143)** | **Mother (n=129)** | **Total (n=272)** | **Father (n=188)** | **Mother (n=177)** | **Total (n=366)** |
| PROTECTION AGAINST INFECTIONS/DISEASES (NET) | 9% | 6% | 8% | 8 | 4 | 6 |  | 1% | 2% | 1% | 1% | 5% (D) | 2% |
| Offers protection/ 100% protection | 1% | - | 1% | 2% | - | 1% |  | - | - | - | - | 1% | * |
| Protects against diseases/future diseases/free from disease | 1% | 1% | 1% | 3% | 2% | 2% |  | - | - | - | - | 2% (D) | 0 |
| Protects them from infection/future infections//less risk of infection | 3% | 4% | 4% | 2% | 1% | 1% |  | - | 1% | * | 1% | - | * |
| Protects against STIs/STDs/protects them when sexually active | 1% | 1% | 1% | 1% | 2% | 1% |  | - | - | - | - | 2% (D) | 0 |
| Protects them against HIV/AIDS | 2% | - | 1% | 1% | 2% | 1% |  | 1% | - | * | - | - | - |
| Coincides with immunization/covers immunization | 2% | 1% | 1% | - | - | - |  | - | - | - | - | - | - |
| Other protection against infections/diseases | - | - | - | - | - | - |  | - | 1% | * | - | - | - |
| CLEANLINESS (NET) | 3% | 1% | 2% | 6% (E) | - | 3% |  | - | - | - | - | - | - |
| He will be clean/grow up clean/clean penis | 3 % (E) | - | 2% | 5% (E) | - | 2% |  | - | - | - | - | - | - |
| Hygienically better | - | 1% | * | 1% | - | * |  | - | - | - | - | - | - |
| HEALING (NET) | 44% | 48% | 46% | 24% | 36% (D) | 30% |  | 17% | 20% | 18% | 5% | 11% (D) | 8% |
| Heals fast/faster | 41% | 44% | 42% | 22% | 33% (D) | 28% |  | 12% | 14% | 13% | 5% | 10% (D) | 7% |
| Heals safely/easily/properly/less scarring | 3% | 6% | 5% | 2% | 3% | 2% |  | 6% | 6% | 6% | - | 1% | * |
| PAIN (NET) | 39% | 41% | 40% | 30% | 29% | 30% |  | 14% | 18% | 16% | 9% | 15% | 12% |
| He won't remember/know the pain/won't remember anything | 9% | 9% | 9% | 6% (E) | 0 | 0 |  | - | - | - | 1% | - | * |
| Less pain/won't suffer as much | 23% | 30% | 27% | 21% | 26% | 23% |  | 4% | 7% | 6% | 3% | 6% | 5% |
| He won't feel the pain/no pain/won't suffer | 7% | 5% | 6% | 2% | 2% | 2% |  | - | 1% | * | - | - | - |
| He's able to withstand the pain/easier to endure the pain | 1% | - | * | 1% | 1% | 1% |  | 9% | 9% | 9% | 5% | 7% | 6% |
| It's painful/too painful/babies can't handle the pain | - | - | - | 1% | - | 1% |  | 1% | 2% | 1% | 1% | 1% | 1% |
| OTHER PERSONAL BENEFITS (NET) | 28% | 27% | 28% | 37% | 31% | 34% |  | 63% (E) | 50% | 57% | 59% | 45% | 52% |
| His skin is soft/fresh/not too hard | - | 1% | * | 9% | 7% | 8% |  | 1% | 2% | 1% | 0 | 3% (E ) | 2% |
| His skin/penis is strong/bigger | - | - | - | - | - | - |  | 1% | 2% | 1% | 3% | 2% | 3% |
| He is strong/stronger/cells/immune system are strong | 2% | - | 1% | - | - | - |  | 1% | 2% | 1% | 1% | 3% | 2% |
| He'll know what's happening/have full knowledge of what and why it's happening/able to reason/understand | 1% | 1% | 1% | - | 1% | * |  | 6% | 3% | 4% | 13% (E) | 6% | 10% |
| He won't know what's happening/has no knowledge of procedure | 2% | 2% | 2% | 3% | 4% | 3% |  | 1% | - | 1% | 2% | 2% | 2% |
| He's not sexually active/before he's sexually active | 6% | 2% | 4% | 9% (E) | 2% | 6% |  | 20% (E) | 5% | 12% | 20% (E) | 10% | 15% |
| He is/starting to be/sexually active/has sexual benefits | - | 1% | * | 1% | - | * |  | 7% | 9% | 8% | 2% | - | 1% |
| He is smart at an earlier age/will grow up smart | - | 2% | 1% | - | - | - |  | 1% | - | 1% | - | - | - |
| He can make his own decisions/can be involved in the decision making | 1% | 1% | 1% | 1% | 1% | 1% |  | 21% (E) | 10% | 16% | 3% | 3% | 3% |
| He won't have to make a decision later in life/the parents decide/he will grow up circumcised | 9% | 5% | 7% | 6% | 3% | 4% |  | - | - | - | 1% | 2% | 1% |
| They can't be discouraged by others/they can't discourage others | 3% | - | 1% | 1% | - | * |  | - | - | - | - | - | - |
| He can be influenced/discouraged by others/open to peer pressure/new ideas | - | 1% | * | 1% | - | 1% |  | 1% | 2% | 2% | - | - | - |
| They can communicate (better than babies) talk about it/the pain/rather than just crying | - | - | - | - | - | - |  | 2% | 2% | 2% | 4% | 2% | 3% |
| Parental care/I/other people will be able to care for child/manage(clean) his wound/easily | 9% | 13% | 11% | 3% | 8% (D) | 5% |  | 1% | 2% | 1% | 1% | 3% | 2% |
| He will be able to care for himself/his wound/keep himself clean/follow instructions | - | - | - | - | 1% | * |  | 3% | 12% (D) | 7% | 6% | 7% | 7% |
| Easier/easier to care for/manage/clean/care for the wound/good care | 1% | 1% | 1% | 2% | 4% | 3% |  | 3% | 3% | 3% | 1% | 1% | 1% |
| He doesn't have feelings/strong feelings/less sensitive/aware/less fear | - | 1% | * | 2% | 2% | 2% |  | - | - | - | 4% (E) | 1% | 2% |
| There will be less embarrassment/shyness | NA | NA | NA | NA | NA | NA |  | 1% | - | * | 1% | 1% | 1% |
| Less mobile | - | 1% | * | 1% | - | * |  | - | - | - | - | - | - |
| OTHER MISCELLANEOUS COMMENTS (NET) | 9% | 9% | 9% | 14% (E) | 4% | 9% |  | 17% | 23% | 20% | 23% | 27% | 25% |
| Other personal benefit | 1% | - | * | - | 1% | 1% |  | - | - | - | - | 2% | 1% |
| Less/no complications/risk/it's safer | 2% | 2% | 2% | 1% | 1% | 1% |  | 3% | 2% | 2% | 1% | 1% | 1% |
| Its advised in the bible/done in our culture/tradition | 1% | 2% | 2% | 1% | - | 1% |  | 4% | 3% | 4% | - | - | - |
| Its most appropriate age/older/still young | 7% | 3% | 5% | 9% (E) | 3% | 6% |  | 9% | 17% | 13% | 22% | 25% | 23% |
| No specific age/any age | - | - | - | - | - | - |  | - | - | - | - | - | - |
| Other | - | 2% | 1% | 3% (E) | 1% | 2% |  | 1% | 2% | 1% | 1% | 2% | 1% |
| OTHER NEGATIVE MISCELLANEOUS COMMENTS (NET) | 2% | - | 1% | - | - | - |  | 1% | 2% | 1% | 1% | 1% | 1% |
| I don't believe in it/won't be doing it any age/unnecessary | 1% | - | * | - | - | - |  | - | - | - | - | - | - |
| There is no ideal age | - | - | - | - | - | - |  | - | - | - | - | - | - |
| It's dangerous/risky/unsafe/scared for the child | - | - | - | - | - | - |  | - | - | - | 1% | - | * |
| Unchristian/against God/my religion/not our culture | - | - | - | - | - | - |  | - | 1% | * | - | - | - |
| Other negative comments | 1% | - | * | - | - | - |  | 1% | 1% | 1% | - | 1% | * |
| NONE/NO ANSWER | - | - | - | 1% | 1% | 1% |  | - | 1% | * | 2% | - | 1% |
| DON´T KNOW | - | - | - | 3% | 5% | 4% |  | 4% | 4% | 4% | 6% | 6% | 6% |
| *NA = not applicable; *, samples size too small; E, statistically significant higher value among males compared to females; D Statistically significant higher value among females compared to males* | | | | | | | | | | | | | |
|  |  |  |  |  |  |  |  |  |  |  |  |  |  |
