# Supplementary material for: Attitudes and decision-making about early-infant versus early-adolescent male circumcision: Demand-side insights for sustainable HIV prevention strategies in Zambia and Zimbabwe
Source: PLoS One. 2017 Jul 27;12(7):e0181411. doi: 10.1371/journal.pone.0181411 (PMC5531536; doi:10.1371/journal.pone.0181411)
Supplement: S1 File — (PDF) [file pone.0181411.s002.pdf]

Introduction to antenatal/neonatal discussion guide [INTERVIEWERS ONLY]

- The purpose of this guide is to understand the role of antenatal and neonatal healthcare professionals in advising parents about circumcision
- The guide also is designed to explore attitudes towards VMMC by healthcare workers and whether there is a preference towards circumcising baby boys, adolescent boys or adult men
- This qualitative phase is exploratory to inform effective development of the market-representative quantitative survey among parents, thus the sampling ensures a broad coverage of districts.

	Copperbelt (Kitwe)	Eastern (Katete)	Lusaka (Lusaka)	Northern (Kasama)	Southern (Kalomo)	Central (Kabwe)	Total
Antenatal/ Neonatal Sample	3	3	3	3	3	3	**18**

Objectives:-

- Identify and assess perceptions and roles, motivations and mechanisms of influence of healthcare providers: Antenatal and neonatal healthcare workers
- Identify interest and role in promoting EIMC/EAMC to parents from healthcare workers

VMMC Antenatal/ Neonatal Interview

Discussion Guide Outline –Final

August 2014

Introduction to Antenatal/ Neonatal discussion guide [TO READ TO RESPONDENTS]

Today, we're going to talk about health and children. There are no right or wrong answers, and I personally am not looking for any response other than your own truth and how you, specifically, feel. Everything you say will be held in the strictest confidence and you will not be judged by any of your responses.

- Everything said in the interview is completely confidential- similarly we would ask you not to discuss this interview with other people after it has ended
- The interview will take about 40 minutes
- There are no right or wrong answers to the questions…we are only interested to hear what you think
- As an independent market research organization, we are committed to ensuring full confidentiality for you in these questions. We will NOT share your answers to these questions – we will only be reporting the results of this discussion together with those of many other people we are interviewing, so what you share will not be identified as your individual thoughts or experiences, and your name will not be used in our reports.
- At times, the discussion will cover personal and sensitive topics such as circumcision, but please be honest and open when sharing your thoughts and experiences on these topics as it is important for us to understand your actual opinion.
- You have the right to withdraw from the interview at any point

1. **INTRODUCTIONS: [5 min.]**

Objective: To build rapport between the respondent and interviewer. To gain context of the healthcare workers place of work and their role specifically. To understand their point of view on HIV

CLINIC CONTEXT

a. To start, tell me a bit about your working role- for example your title and your main responsibilities
    i. [INTERVIEWER NOTE: IF NOT MENTIONED ASK: Specifically tell me about your role in AnteNatal Care/ NeoNatal care]
    ii. Thinking more specifically about your work setting, what services does your facility offer? [PROBE: AnteNatal and NeoNatal]
    iii. How many people do you see a month?
    iv. What kind of people come to see you for support?
    v. For what reasons do people come to you for support?
    vi. At what times specifically do people come for services and what do they need at each time?

HIV CONTEXT

a. Now I'd like to hear your thoughts on HIV. What proportion of people who come to your clinic have HIV?
b. How aware are your patients about HIV?
c. What concerns do your patients have about HIV?
d. What, if any, actions do they take to prevent HIV?
e. What do you see as the most effective HIV prevention method for your community specifically?

2. Role of antenatal/ neonatal as an influencer[5 mins]

Objective: To understand more about the counselling interactions between healthcare workers and parents. To uncover perceptions around who are the other main influencers around child birth in the community

- When expectant or new mothers come to your clinic who, if anyone, do they come with?
    - What percentage of your time is spent advising new parents or those about to have a baby?
    - Typically, what issues do you counsel new parents/ those about to have a baby about?
    - What kind of questions do you get asked about by new parents/ those about to have a baby?
    - Where else do these people get advice about their new born/ soon to be born baby?

- Who in the community (including family members) is influential in advising parents about their new borns/soon to be born babies?
    - What makes them influential?

- How influential are the grandparents (parents parents)? What makes them influential/ non influential?

- How influential are the about friends of parents? What makes them influential/ non influential?

- What about pastors or minister at their church? What makes them influential/ non influential?

- If a father and mother who were expecting their first child came to you and asked for health advice for their new born **baby boy**, what would you advise? Why?
    - Where should these expectant parents get health advice for their new born baby boy?

- Has the advice you give new fathers and mothers changed over time?
    - If so, how?
    - Why?

- What are the main community customs and traditions on having children?
    - [INTERVIEWER INSTRUCTIONS:- PROBE DURING PREGNANCY
        - IMMEDIATELY AFTER BIRTH
        - IN THE FIRST YEAR
        - AFTER THE FIRST YEAR (RAISING A CHILD)]
    - Are they different for boys and girls?

- How important is it to uphold community customs and traditions around raising a child?

3. ATTITUDES TO VMMC [10 MIN]

Objective: To understand the VMMC sustainability drive from front line healthcare workers point of view. To gain insight into the conversations parents and healthcare workers are having with each other regarding infant/ adolescent circumcision. To gain a snapshot of the healthcare workers personal beliefs regarding VMMC

- Now I'd like to get your thoughts on circumcision- what are your thoughts on it? Just think about those that come to mind first when you think about circumcision.

- How familiar are you with Voluntary Male Medical Circumcision often referred to as VMMC? What is your understanding of VMMC?
    - Who typically is a candidate for VMMC?
    - What are your thoughts on circumcising baby boys?
        - What are the advantages of circumcising baby boys?
        - Are there any negatives about circumcising baby boys?

- How would you feel if you were asked to provide a circumcision service for baby boys?
    - What do you think about carrying out the procedure?
    - How equipped in your clinic to offer this service?
    - What capacity does your clinic have to potentially offer a circumcision service for baby boys?

- Do you discuss the topic of circumcision with expectant parents/ new parents?
    - [INTERVIEWER NOTES: IF YES ASK] Do you raise the topic with them or do they usually raise it with you? What are the people you discuss circumcision with like? What is discussed?
    - [INTERVIEWER NOTES: IF NO] What are the reasons why you do not discuss circumcision?

- What questions do parents have about circumcision if any?

- What other places do you think parents hear about circumcision?
    - How accurate do you think this information is

- Based on your experiences how do you think the wider community feel about circumcision?

- Where would people go to circumcise baby boys?
    - What would be their reasons for circumcising their baby boys?
    - Why do some people not want to circumcise baby boys?
    - What types of parents, in your opinion circumcises their baby boys?

- Where would people go for circumcision advice?
    - What about to have the procedure done?
    - How does the procedure work? [PROBE: Before, During, After]
    - How much does it cost to get circumcised?
    - Do you feel that this is a reasonable cost for you patients/ community?

- In your opinion, when would be the best time for a male to be circumcised:
    - As a baby (less than 2 months)
    - As a young adolescent (aged 10-14)
    - As a man (aged 18+)
    - Never

[INTERVIEWER NOTES: PROBE FOR REASONS WHY]

4. PERCEIVED COMMUNITY FEELINGS TO VMMC [5 MIN]

Objective: To gain an understanding of perceived community feeling towards VMMC and specifically with baby boys/ infant boys

- Who in your opinion would have the biggest influence in general on the parents decision to circumcise their baby boy?
    - Who else is influential?
- What, if anything, do pastors/ ministers and other community elders say about circumcising baby boys?
- What about other healthcare workers?
- If the pastors/ ministers in the community advised parents against circumcising their boys but healthcare workers advised parents to circumcise their boys how would people react? Why?
- Who do you think would be the biggest supporter of circumcising baby boys in the community?
    - Why?
- Who in the community do you think would be most against circumcising baby boys?
    - What would make them change their mind?

5. MAKING THE DECISION TO CIRCUMCISE [10 MIN]

Objective: To encourage respondents to think of the ideal platforms in which the sustainability message can be conveyed. To ascertain what parents need to know before deciding to circumcise their boys.

- What do people need to know before they could make a decision about whether to circumcise their baby boy or not?
    - Where do they find out this information from?
- Is it better to circumcise baby boys or teenage boys? Why?
    - Is it better to circumcise baby boys or adult men? Why?
- What would it take for people to decide to circumcise their baby?
    - How important is it for people that their parents approve?
    - What about their grand parents?
    - What about elders in the wider community?
- How aware do you think parents in your community are about the benefits of circumcising baby boys?
    - What about circumcising adolescent boys?

- How aware do you think parents in your community are about how the procedure works?
  - Where do parents become aware of how the procedure works?
  - Where do you think should be the ideal place for parents to become aware of how the procedure?

- And how aware are parents in general of the facilities (clinics/hospitals) which carry out circumcision?
  - How do parents become aware of these facilities?

OK now please think about how to best encourage parents to consider circumcising baby boys:

- Who should be responsible for encouraging parents to consider circumcising baby boys? Why?
  - Where should this happen? Why?
  - What should they say? Why?

- What are the challenges which may be faced by the approach you have just outlined to encourage parents to circumcise baby boys?

Now thinking about adolescent circumcision…

- Who should be responsible for informing parents about the benefits of circumcising baby boys to help them when considering circumcising baby boys? Why?
  - Where are the different places this should this happen? When? Why? [INTERVIEWER NOTE: PROBE VARIOUS LOCATIONS]
  - What should they say? Why?

- What are the challenges which may be faced by the approach you have just outlined to encourage parents to circumcise adolescent boys?

- Do people trust the healthcare facilities to do the best for them and their children?
  - Why? Why not?

- Do people trust pastors/ ministers to do the best for them and their children?
  - Why? Why not?

- Do people trust community elders to do the best for them and their children?
  - Why? Why not?

6. WRAP UP [5 MIN]

Objective: To identify any gaps which healthcare workers perceive that stop them from being able to advise parents on circumcision

Finally…

- What resources do you currently have to help you give full advice on circumcising baby boys?

- What more do you need to help support you in advising on circumcising baby boys ?

- Do you have any other comments on circumcision amongst baby boys and adolescent boys which are not covered?
