# Supplementary material for: Attitudes and decision-making about early-infant versus early-adolescent male circumcision: Demand-side insights for sustainable HIV prevention strategies in Zambia and Zimbabwe
Source: PLoS One. 2017 Jul 27;12(7):e0181411. doi: 10.1371/journal.pone.0181411 (PMC5531536; doi:10.1371/journal.pone.0181411)
Supplement: S2 File — (PDF) [file pone.0181411.s003.pdf]

Introduction to Provider discussion guide [INTERVIEWERS ONLY]

- The purpose of this guide is to understand the experiences trained providers of EIMC have in counselling and servicing parents and their infants for MC
- The guide also is designed to explore attitudes towards VMMC by healthcare workers and whether there is a preference towards circumcising baby boys or adolescent boys from the community
- Due to the relatively low number of trained EIMC providers in Zambia, the sampling acknowledges that only a few trained EIMC providers exist in Zambia and a number of these will be interviewed.

	Copperbelt (Kitwe)	Lusaka (Lusaka)	Total
**Providers**	3	5	**8**

Objectives:-

- Identify circumcision knowledge gaps amongst parents
- Understand perceptions about circumcision from interactions between providers and parents
- Identify barriers parents are facing around circumcision and understand how representatives of the provision system help parents overcome these barriers

VMMC EIMC Provider Interview

Discussion Guide Outline –Final

August 2014

Introduction to Provider discussion guide [TO READ TO RESPONDENTS]

Today, we're going to talk about circumcision and children. There are no right or wrong answers, and I personally am not looking for any response other than your own truth and how you, specifically, feel. Everything you say will be held in the strictest confidence and you will not be judged by any of your responses.

- Everything said in the interview is completely confidential- similarly we would ask you not to discuss this interview with other people after it has ended
- The interview will take about 40 minutes
- There are no right or wrong answers to the questions…we are only interested to hear what you think
- As an independent market research organization, we are committed to ensuring full confidentiality for you in these questions. We will NOT share your answers to these questions – we will only be reporting the results of this discussion together with those of many other people we are interviewing, so what you share will not be identified as your individual thoughts or experiences, and your name will not be used in our reports.
- At times, the discussion will cover personal and sensitive topics such as circumcision, but please be honest and open when sharing your thoughts and experiences on these topics as it is important for us to understand your actual opinion.
- You have the right to withdraw from the interview at any point

1. INTRODUCTIONS: [5 min.]

Objective: To build rapport between the respondent and interviewer. To gain context of the providers place of work, the types of procedures they do and with whom. To understand providers role specifically

- To start, tell me a bit about your working role- for example your title and your main responsibilities

- Thinking more specifically about your work setting, what services do you offer within the clinic? [INTERVIEWER NOTE: PROBE ALL SERVICES]

- [INTERVIEWER NOTE: IF NOT MENTIONED ASK] Specifically tell me about your role in carrying out the circumcision procedure

- Who else is present during the circumcision procedure? [INTERVIEWER NOTES: PROBE OTHER CLINICAL STAFF AND THEIR ROLE, FAMILY MEMBERS, FRIENDS, OTHER PEOPLE FROM THE COMMUNITY]

- How many men or boys are circumcised every month in your clinic?
  1. What proportion of those are baby boys?
  2. And what about adolescent boys? (ages 10-14)
  3. And finally, what proportion are adult men? (ages 15+)

- Please explain to me how the circumcision procedure works with a baby boy from beginning to finish:
  - Who is in the room?
  - How is the baby prepared?
  - What is the procedure?
  - How is the foreskin discarded?
  - What do you tell the parents about looking after the baby after the circumcision?

- How satisfied do you think the parents of the boys are with the methods of circumcision you use? Why?

For the rest of the interview, we will only talk about the circumcision of baby boys and adolescent or young boys

- What age range are the baby boys that are circumcised within your clinic?

- And what age range are the adolescent or young boys you circumcise within the clinic?

- For the rest of the interview we will refer to adolescent boys as being between the ages of 10 and 14. We will refer to baby boys as babies under the age of 2 months

2. COUNSELLING VMMC/ CIRCUMCISION: [10 min.]

Objective: To gain context of how long providers spend counselling parents, where and when. To gain insight into the conversations providers and parents have around circumcision

OK, now thinking about all the different times you speak to parents about circumcision…

- In what situations do you speak to parents about circumcision?
  - Is it when they have shown an interest in circumcising their boy or do you present/suggest the option to them?
  - Is it different for parents of new born babies compared to adolescent boys?
  - When you speak to parents have they already made the decision to circumcise their boys?
  - Are there any other times when you speak to parents about baby health apart from circumcision? When and why?

- Where do these discussions take place? [INTERVIEWER NOTES: PROBE IF NOT MENTIONED- IN CLINIC/ HOSPITAL, IN HOMES, IN CHURCH, IN COMMUNITY CENTRES, OTHER]
  - Does this differ for babies compared to adolescent boys?

- Apart from parents, are there any other people you speak to about circumcising a baby boy or young boy? [INTERVIEWER NOTES: PROBE IF NOT MENTIONED- GRAND PARENTS, FRIENDS OF PARENTS, OTHER FAMILY MEMBERS, COMMUNITY ELDERS, MINISTERS OR PASTORS, OTHERS]

- Imagine you are meeting parents who are expecting a baby boy and you are telling them about circumcision. They have not considered circumcision before and you are meeting them at a social event. What would you say?
  - From your point of view, is it better to circumcise baby boys or young adolescent boys? Why?
  - What about from the parents' point of view? What are their reasons?

- Who else influences the parents' decision to circumcise a baby boy? In what way do they influence the decision?
  - What about an adolescent boy? In what way do they influence the decision?

- Please tell me in full how you would explain the circumcision procedure to parents of a <u>baby boy</u>:
  - [INTERVIEWER NOTES: IF PRE-PROCEDURE REQUIRMENTS NOT MENTIONED ASK:] what, if anything, do you tell the parents about what needs to be done before the actual procedure?

  - And what about after the procedure? How do you explain aftercare for the baby once he has been circumcised?

- What questions do parents have specifically about each part of the procedure?
  - How do you answer these questions?

- Please tell me in full how you would explain the circumcision procedure to parents of a young or adolescent boy.
  - How do you explain what will happen during the procedure?
  - And what about after the procedure? How do you explain aftercare for the boy once he has been circumcised?

- What questions do parents have specifically about each part of the procedure?
  - How do you answer these questions?

3. PARENTS UNDERSTANDING OF VMMC/ CIRCUMCISION [10 min.]

Objective: To understand the knowledge parents have of circumcision when they arrive in the clinic and its source. To identify the fears and concerns of parents who are deciding to circumcise their children and the reasons why they decide to circumcise

Now I would like to learn more about parents' understanding of circumcision, the benefits, risks and procedure…

- Apart from you, who else do parents speak to about circumcision? [INTERVIEWER NOTES: ALLOW ADEQUATE TIME FOR SPONTANEOUS ANSWER. PROBE IF NOT MENTIONED: OTHER HEALTHCARE WORKERS- WHO? THEIR PARENTS (GRAND PARENTS), FRIENDS, OTHER FAMILY MEMBERS- WHO? COMMUNITY ELDERS, MINISTERS OR PASTORS, OTHERS]

- What do they hear about circumcision from these other people they speak to?

- What fears and concerns do parents have about circumcision before they come to the clinic?
  - Does this differ for baby boys compared to adolescent boys? In what way(s)?

- What are the reasons why some parents make the decision to circumcise their baby boys? Please list all the reasons you are aware of:
  - What is the biggest reason that encourages parents to make the step to circumcise their baby boy? Why?

- What are the reasons why other parents do not want to circumcise their baby boys? Again, please list all reasons as you are aware of.
  - What is the biggest reason why some parents don't want to circumcise their baby boy? Why?

I want you think about the differences in parents' views on circumcising babies and young adolescents…

- How, if at all, do the reasons why parents decide to circumcise their baby boy differ to those that cause them to circumcise their adolescent boy?

  And how, if at all, do the reasons why parents decide to not circumcise their baby boy differ to the reasons why they don't circumcise their adolescent boy?

- When parents have made the decision to circumcise and they come to the clinic, what do they know about circumcision?
  - What questions do they have at this stage?
  - How does this differ for babies compared to adolescent boys?

4. ENCOURAGING 'SUSTAINABILITY': [10 min.]

Objective: To encourage respondents to think of the ideal platforms in which the sustainability message can be conveyed

Now I would like you to think about how to best encourage parents to consider circumcising their boys, babies and adolescents.

- But first, in your opinion what do you think the benefits actually are about circumcising baby boys?
  - What about compared to adolescent boys?

- How aware do you think parents in your community are about the benefits of circumcising baby boys?
  - What about circumcising adolescent boys?

- How aware do you think parents in your community are about how the procedure works?
  - Where do parents become aware of how the procedure works?
  - Where do you think should be the ideal place for parents to become aware of how the procedure?

- And how aware are parents in general of the facilities (clinics/hospitals) which carry out circumcision?
  - How do parents become aware of these facilities?

- Do you think that parents would rather circumcise baby boys or adolescent boys? Why?

OK now please think about how to best encourage parents to consider circumcising baby boys:

- Who should be responsible for informing parents about the benefits of circumcising baby boys to help them when considering circumcising baby boys? Why?

  - Where are the different places this should this happen? When? Why? [INTERVIEWER NOTE: PROBE VARIOUS LOCATIONS]
  - What should they say? Why?

- What are the challenges which may be faced by the approach you have just outlined to encourage parents to circumcise baby boys?

Now thinking about adolescent circumcision…

- Who should be responsible for informing parents about the benefits of circumcising baby boys to help them when considering circumcising adolescent boys? Why?
  - Where are the different places this should this happen? When? Why? [INTERVIEWER NOTE: PROBE VARIOUS LOCATIONS]
  - What should they say? Why?

- What are the challenges which may be faced by the approach you have just outlined to encourage parents to circumcise adolescent boys?

- Do people trust the healthcare facilities to do the best for them and their children?
  - Why? Why not?

- Do people trust pastors/ ministers to do the best for them and their children?
  - Why? Why not?

- Do people trust community elders to do the best for them and their children?
  - Why? Why not?

5. WRAP UP: [5 min.]

Objective: To identify any gaps which providers perceive that stop them from being able to advise parents on circumcision

Finally…

- What resources do you currently have to help you give full advice on circumcising baby boys?
  - What about adolescent boys?

- What more do you need to help support you in advising on circumcising baby boys?
  - What about adolescent boys?

- Do you have any other comments on encouraging circumcision amongst baby boys and adolescent boys?
