# Supplementary material for: Attitudes and decision-making about early-infant versus early-adolescent male circumcision: Demand-side insights for sustainable HIV prevention strategies in Zambia and Zimbabwe
Source: PLoS One. 2017 Jul 27;12(7):e0181411. doi: 10.1371/journal.pone.0181411 (PMC5531536; doi:10.1371/journal.pone.0181411)
Supplement: S3 File — (PDF) [file pone.0181411.s004.pdf]

Introduction to Grand Parents discussion guide [INTERVIEWERS ONLY]

- The purpose of this guide is exploratory- to understand the role and influence of grandparents on parents feelings towards circumcising their children
    - These interviews will identify the perceptions, motivations and roles elders and grandparents have in MC, to understand their point of view and how this distils into the wider family and community. This qualitative phase is exploratory to inform effective development of the market-representative quantitative survey among parents, thus the sampling ensures a broad coverage of districts.

- Each Grandmother (or Grandmother figure) and Grandfather (or Grandfather figure) are interviewed separately from each other

	Copperbelt (Kitwe)	Eastern (Katete)	Lusaka (Lusaka)	Northern (Kasama)	Southern (Kalomo)	Central (Kabwe)	Total
Paternal Grandfathers	2	1	3	1	1	1	**9**
Paternal Grandmothers	2	1	3	1	1	1	**9**

Objectives:-

- Assess perceived role of culture, religion and orientation to traditional MC in decision-making; identify parents' reliance on community leaders in communicating importance of EIMC/EAMC and appropriate age for circumcising non sexually active boys (NSABs)
- Identify and assess the father/ mother role in decision making and underlying knowledge and beliefs that drive willingness to seek MC for his/ her grandson
- Identify and assess perceptions and roles, motivations and mechanisms of influence of grandparents

VMMC Grand Parent Interview

Discussion Guide Outline –Final

August 2014

Today, we're going to talk about health and children. There are no right or wrong answers, and I personally am not looking for any response other than your own truth and how you, specifically, feel. Everything you say will be held in the strictest confidence and you will not be judged by any of your responses.

- Everything said in the interview is completely confidential- similarly we would ask you not to discuss this interview with other people after it has ended
- The interview will take about 40 minutes
- There are no right or wrong answers to the questions…we are only interested to hear what you think
- As an independent market research organization, we are committed to ensuring full confidentiality for you in these questions. We will NOT share your answers to these questions – we will only be reporting the results of this discussion together with those of many other people we are interviewing, so what you share will not be identified as your individual thoughts or experiences, and your name will not be used in our reports.
- At times, the discussion will cover personal and sensitive topics such as circumcision, but please be honest and open when sharing your thoughts and experiences on these topics as it is important for us to understand your actual opinion.
- You have the right to withdraw from the interview at any point.

1. INTRODUCTIONS: [5 min.]

Objective: To build rapport between the respondent and interviewer. To encourage the respondent to think about their grandchildren's health and also assess their understanding of HIV

FAMILY CONTEXT

- To start, tell me a bit about your family.
    - i. Who do you live with?
    - ii. How many children do you have?
    - iii. How many grand children do you have? Are they girls or boys?
    - iv. How old are they?
    - v. Are you looking after or responsible for any children which are not yours? How old are they?
    - vi. Generally, what aspirations do you have for your grandchildren when they grow-up?

HEALTHY FAMILY CONTEXT

a. Now I would like you to think about your grandchildren's health. What is most important to you when thinking about your grandchildren's health. Please name as many things as you like [INTERVIEWER INSTRUCTIONS:- PROBE FOR ATLEAST 3]
b. What is the biggest concern you have about your grandchildren's health?

- How do health concerns about adults differ to health concerns about grandchildren?

HIV CONTEXT

a. Now I'd like to hear your thoughts on HIV. What do you know about HIV?

b. How do you feel about HIV? Tell me a few thoughts you have on HIV, in general. [INTERVIEWER INSTRUCTIONS: PROBE FOR THE LIST BELOW]
    a. Effects of HIV on family
    b. Effects of HIV on community
    c. Causes of HIV
    d. Treatment for HIV
    e. Effects of HIV on relationships

2. PERCEIVED ROLE AS INFLUENCER [10 mins]

Objective: To understand the different influencers in the community around raising children and where the grandparents see their role. To explore thoughts and feelings towards different people in the community who may offer advice on raising children. To understand community customs and traditions around having children

- Where do new or expecting parents in your community go to learn about looking after a child's health?
  - Who are the important people in the community who teach others about having children?
  - What makes them important?

- What type of help do you give your children with looking after their children?
  - How regularly do you help your children look after their children?

- Who else apart from yourself, helps your children look after your grand children?
  - What help do they provide?

- Thinking back to your **first grandchild**, what advice did you give your son/ daughter about looking after their baby's health?

[INTERVIEWER NOTE: IF THE RESPONDENT HAS MORE THAN 1 GRANDCHILD, PLEASE ASK THE QUESTION BELOW]

  - Has this advice changed for your other grandchildren?

- How important is it that your children follow advice about looking after your grandchildren's health from healthcare workers? Why/ why not?

- How important is it that your children follow advice about looking after your grandchildren's health from pastors/ ministers? Why/ why not?

- How important is it that your children follow advice about looking after your grandchildren's health from you? Why/ why not?

- Who do you trust most to give advice on looking after children's health? Why?

- Are there any people who you trust less to advise your children on looking after their children's health? What makes them less trustworthy?

- Imagine parents who are expecting or who have just had their first child who is a boy, are there any differences in where they should get advice compared to if it was a girl?

  - What advice would you give this couple?

- What are the main community customs and traditions on having children?
  - [INTERVIEWER INSTRUCTIONS:- PROBE DURING PREGNANCY
    - IMMEDIATELY AFTER BIRTH
    - IN THE FIRST YEAR
    - AFTER THE FIRST YEAR (RAISING A CHILD)]
  - Are they different for boys and girls?

- How important is it to uphold community customs and traditions?
- Are some customs more important to uphold than others? Which ones and why?
- Are you aware of any community customs or tradition which have changed ? How did it change? Why?
- Are you aware of any advice given by healthcare workers which is different to community customs and traditions?
  [INTERVIEWER INSTRUCTIONS:-IF ANSWER IS YES ASK:]
    - What is this advice?
    - What is your reaction?
    - What do other people in the community think

3. ATTITUDES TO VMMC [10 MIN]

Objective: To explore Grandparent awareness and understanding of circumcision. To identify and explore feelings towards sources of information about circumcision. To uncover a range of Grandparental attitudes towards VMMC

- Now I'd like to get your thoughts on circumcision- what are your thoughts on circumcision? Just think about those that come to mind first when you think about circumcision.
- What, if anything, have you heard about circumcising baby boys (less than 2 months old)? Please tell us as much as possible.
    - Where did you hear this information?
    - To what extent do you believe this?
- What, if anything, have you heard about circumcising young or adolescent boys? Please tell us as much as possible.
    - Where did you hear this information?
    - To what extent do you believe this?
    - Do boys under the age of 10 get circumcised?
        - Why?
        - How old are these boys?
- What, if anything, have you heard about circumcising adult men (ZAMBIA: over the age of 15)? Please tell us as much as possible.
  Where did you hear this information?
    - To what extent do you believe this?
- What would be your reaction if someone were to recommend that you circumcise your baby grandson?
- Would your reaction be different if someone were to recommend that you circumcise your baby grandson when he is a young adolescent (10-14 years)? Why?
- Who would you trust to talk to about circumcision? Why?
    - Who would you not trust? Why?
- What do you know about parents who circumcise their baby boys?

    - What are their reasons?

- Why do some people not want to circumcise baby boys?

- Do (any) men in your community get circumcised?

- Where would people go to have the circumcision procedure done?
    - How does the procedure work? [INTERVIEWER INSTRUCTIONS:- PROBE: BEFORE, DURING, AFTER]
    - How much does it cost to get circumcised?
    - In your opinion is this a reasonable price?

[INTERVIEWER NOTES: IF HIV NOT MENTIONED ABOVE THEN ASK QUESTION BELOW]

- Some people say that circumcising males will help prevent HIV and other sexual health diseases. What have you heard about this?
    - Who have you heard talk about this and what did they say? [INTERVIEWER NOTES: PROBE POSITIVE ARGUMENTS AND NEGATIVE]
    - Do you think men in your community get circumcised to prevent HIV?

4. PERCEIVED COMMUNITY FEELINGS TO VMMC [10 MIN]

Objective: To explore the role of community influencers in promoting or detracting from the drive to upscale circumcision amongst infants/ adolescents. To uncover the impact of certain community influencers compared to others

- I'm going to name some people in your community and ask you whether they have said anything to you about circumcising baby boys:
    - Pastors/ ministers
    - Community elders
    - Healthcare workers
    - Your partner
    - Your friends

[INTERVIEWER INSTRUCTIONS:- IF THE ANSWER IS YES TO ANY OF THE ABOVE, PLEASE ASK WHAT WAS SAID]

- Do you feel there is a difference in opinions towards male circumcision between your generation and your children's generation? [INTERVIEWER NOTES: IF YES ASK WHY]

- If the religious leader in your community advised parents against circumcising their boys but healthcare workers advised parents to circumcise their boys how would people react (which advice would they follow)? Why?

- Who do you think would be the biggest supporter of circumcising baby boys from people in the community?
    - What would they say?
    - Why?

- And who do you feel would be most against circumcising baby boys from people in the community?
    - What would they say?
    - What would make them change their mind?

5. MAKING THE DECISION TO CIRCUMCISE [5 MIN]

Objective: To understand Grandparental attitudes to circumcising infants vs adolescents vs adult men

- What would people need to know before they could make a decision about whether to circumcise their baby boy or not?
    - Where would they find out this information?

- What would people need to know before they could make a decision about whether to circumcise their young or adolescent boy or not?
    - Where would they find out this information?

- Do people trust the healthcare facilities to do the best for them and their children?
    - Why? Why not?
