# Supplementary material for: Attitudes and decision-making about early-infant versus early-adolescent male circumcision: Demand-side insights for sustainable HIV prevention strategies in Zambia and Zimbabwe
Source: PLoS One. 2017 Jul 27;12(7):e0181411. doi: 10.1371/journal.pone.0181411 (PMC5531536; doi:10.1371/journal.pone.0181411)
Supplement: S4 File — (PDF) [file pone.0181411.s005.pdf]

Introduction to Parents discussion guide [INTERVIEWERS ONLY]

- The purpose of this guide is exploratory- to uncover a range of potential barriers and drivers to circumcising baby boys (under the age of 2 months), adolescent boys (aged 10-14) and adult men (aged 18+)
- This guide will also explore ‘social constructs’ or cultural barriers to circumcising infants/ adolescents
- The parents which we are speaking to have either just had a baby boy or are expecting a baby (either a baby boy or do not know the sex)
- Each mother and father are interviewed separately from each other

	Copperbelt (Kitwe)	Eastern (Katete)	Lusaka (Lusaka)	Northern (Kasama)	Southern (Kalomo)	Central (Kabwe)	Total
Fathers	3	3	3	3	3	3	**18**
Mothers	3	3	3	3	3	3	**18**

Objectives:-

- Assess awareness and knowledge of benefits and risks of circumcising non sexually active boys (NSABs); identify knowledge gaps about circumcising infants (0-60 days) and boys (10-14 years)
- Identify relative importance of perceived benefits vs. perceived risks on decision-making for circumcising NSABs and trade-offs made among these; assess decision-making models and criteria, including heuristics, among parents for MC of their boys
- Assess perceived role of culture, religion and orientation to traditional MC in decision-making; identify parents’ reliance on community leaders in communicating importance of EIMC/EAMC and appropriate age for circumcising NSABs
- Identify and assess the father/ mother role in decision making and underlying knowledge and beliefs that drive willingness to seek MC for his/ her son; Assess likelihood of a father to circumcise his son whether he is vs. whether he is not circumcised himself
- Assess propensity for parents to prefer EIMC vs. EAMC for their boys

VMMC Parent Interview

Discussion Guide Outline –Final

August 2014

Introduction to Parents discussion guide [TO READ TO RESPONDENTS]

Today, we're going to talk about health and children. There are no right or wrong answers, and I personally am not looking for any response other than your own truth and how you, specifically, feel. Everything you say will be held in the strictest confidence and you will not be judged by any of your responses.

- Everything said in the interview is completely confidential- similarly we would ask you not to discuss this interview with other people after it has ended
- The interview will take about 60 minutes
- There are no right or wrong answers to the questions…we are only interested to hear what you think
- As an independent market research organization, we are committed to ensuring full confidentiality for you in these questions. We will NOT share your answers to these questions – we will only be reporting the results of this discussion together with those of many other people we are interviewing, so what you share will not be identified as your individual thoughts or experiences, and your name will not be used in our reports
- At times, the discussion will cover personal and sensitive topics such as circumcision, but please be honest and open when sharing your thoughts and experiences on these topics as it is important for us to understand your actual opinion
- You have the right to withdraw from the interview at any point

1. INTRODUCTIONS: [5 min.]

Objective: To build rapport between the respondent and interviewer. To encourage the respondent to think about their childrens health and also assess their understanding of HIV

FAMILY CONTEXT

a. To start, tell me a bit about your family.
   i. Who do you live with?
   ii. How many children do you have? How old are they?
   iii. Are you looking after or responsible for any children which are not yours? How old are they?
   iv. What aspirations do you have for your children when they are grown-ups?
   v. Who do other people in your community who are your age live with? How many children do they usually have?

HEALTHY FAMILY CONTEXT

b. Now I would like you to think about your child's health. What is most important to you when thinking about your children's health. Please name as many things as you like [INTERVIEWER INSTRUCTIONS:- PROBE FOR ATLEAST 3]
c. What is the biggest concern you have about your children's health?

- How do health concerns about adults differ to health concerns about children?

HIV CONTEXT

a. Now I'd like to hear your thoughts on HIV. What do you know about HIV?

b. How do you feel about HIV? Tell me a few thoughts you have on HIV, in general. [INTERVIEWER INSTRUCTIONS: PROBE FOR THE LIST BELOW]
   a. Effects of HIV on family
   b. Effects of HIV on community
   c. Causes of HIV
   d. Treatment for HIV
   e. Effects of HIV on relationships

2. KNOWLEDGE ABOUT CHILD HEALTH- IDENTIFYING AND EXPLORING ROLE OF INFLUENCERS [15 mins]

Objective: To understand how parents learn about caring for a new born baby. To assess the role of healthcare workers compared to other influencers in the community. To learn about the importance of community customs in caring for a new born baby

- Thinking back to your first child or when you first realised you were going to become a parent, what were your first feelings?
  - What did you know about looking after a child?

- Who do you trust most to give advice on looking after your child's health? Why?
  - Who do you not trust? What makes them not trustworthy? What types of things do they say?

[INTERVIEWER INSTRUCTIONS: ASK QUESTION BELOW TO ALL THOSE WHO HAVE ALREADY HAD A CHILD]

- For the rest of the interview we will refer to the most recent baby you have had. Think back to the child you have JUST had, tell me all the various places you have gone to, to learn where to best look after your child to ensure that they are healthy?
  [INTERVIEWER INSTRUCTIONS:- ASK QUESTION BELOW TO ALL THOSE WHO HAVE NOT YET HAD ATLEAST ONE CHILD]

- For the rest of the interview we will refer to the baby you will be having shortly. Tell me all the various places you have gone to, to learn where to best look after your future baby to ensure that they are healthy?

[INTERVIEWER INSTRUCTIONS:- ALLOW ENOUGH TIME FOR THOROUGH SPONTANEOUS RESPONSE. PROBE FOR EACH OF THE BELOW IF NOT MENTIONED SPONTANEOUSLY AND SPECIFY WHAT ADVICE WAS GIVEN]

  - Parents
  - Grand parents
  - Wider family
  - Partner
  - Health clinic (specify which?)
  - Community health worker
  - Community chiefs
  - Religious sermons
  - Traditions
  - Any others

- Tell me about the times when you would visit a healthcare worker for help with your baby?
  - Which healthcare workers?
  - Where? [INTERVIEWER INSTRUCTIONS:- PROBE VISITING A HEALTHCARE FACILITY VS WAITING FOR A HEALTHCARE WORKER TO VISIT THE LOCAL VILLAGE OR HOME]
  - For what reasons?
  - What about times when a healthcare worker visits you without a specific need for the baby. What would be their reasons?

- Are there other places where people in your community go to learn about looking after a child's health? What are these places?

- Tell me about a time where you received some advice about your child's health which was particularly useful?

  - Where were you when you received the advice?
  - Who was there?
  - What were you doing
  - Why was the advice useful?
    - Who gave you the advice?
    - What did they say?
    - How did you feel?

- Now tell me about a time where you received advice that was not useful?
  - Why was it not useful?

- If a father and mother who were expecting their first child came to you and asked for health advice for their new born baby boy, what would you advise? Why?
  - Where should a father and mother expecting a first child get health advice for their new born baby boy in particular?

- What are the main community customs and traditions on having children?
  - [INTERVIEWER INSTRUCTIONS:- PROBE DURING PREGNANCY
    - IMMEDIATELY AFTER BIRTH
    - IN THE FIRST YEAR
    - AFTER THE FIRST YEAR (RAISING A CHILD)]
  - Are they different for boys and girls?

- How important is it to uphold community customs and traditions around raising a child?

- Are some customs more important to uphold then other? Which ones and why?

- Are you aware of any community customs or tradition which have changed? How did it change? Why?

- Are you aware of any advice given by healthcare workers which is different to community customs and traditions?
  [INTERVIEWER INSTRUCTIONS:-IF ANSWER IS YES ASK:]
  - What is this advice?
  - What is your reaction?
  - What do other people in the community think?

3. ATTITUDES TO VMMC [15 MIN]

Objective: To explore parent awareness and understanding of circumcision. To identify and explore feelings towards sources of information about circumcision. To uncover a range of parental attitudes towards VMMC

- Now I'd like to get your thoughts on circumcision- what are your thoughts on circumcision? Just think about those that come to mind first when you think about circumcision.

[INTERVIEWER INSTRUCTIONS:- IF INTERVIEWING FATHER ASK:]

- Are you circumcised?

  - [IF YES] What were the reasons why you got circumcised?
  - [IF NO] Do you to get circumcised in the future?

- [INTERVIEWER INSTRUCTIONS:- IF INTERVIEWING MOTHER ASK:]

- Is your partner circumcised?

  - [IF YES] What were the reasons why they got circumcised?
  - [IF NO] Does your partner plan to get circumcised in the future?

- What, if anything, have you heard about circumcising baby boys (less than 2 months old)? Please tell us as much as possible.
  - Where did you hear this information?
  - To what extent do you believe this?

- What, if anything, have you heard about circumcising young boys (aged 10-14 years)? Please tell us as much as possible.
  - Where did you hear this information?
  - To what extent do you believe this?
  - Do boys under the age of 10 get circumcised? Why? How old are these boys?

- What, if anything, have you heard about circumcising adult men (ZAMBIA: over the age of 15)? Please tell us as much as possible.
  Where did you hear this information?
  - To what extent do you believe this?

- What would be your response if someone recommended that you circumcise your baby boy? Why?

- What would be your response if someone recommended that you circumcise your baby boy when he becomes an adolescent (age 10 -14 years)? Why?

[INTERVIEWER INSTRUCTIONS:- ASK BELOW IF HIV PREVENTION NOT ANSWERED IN QUESTION ABOVE]

- Some people say that circumcising males will help prevent HIV and other sexual health diseases. What, if anything, have you heard about this?
  - Who have you heard talk about this and what did they say?

[INTERVIEWER INSTRUCTIONS:- PROBE POSITIVE ARGUMENTS AND NEGATIVE]

- Do (any) men in your community get circumcised?
- Do you think men in your community get circumcised to prevent HIV?

- Who would you trust to talk to about circumcision? Why?
  - Who would you not trust? Why?
  - Which other people might others go to for information or advice on circumcision?

- What do you know about parents who circumcise their baby boys?
  - What are their reasons?

- Why do some people not want to circumcise baby boys?

- Where would people go to have the circumcision procedure done?
  - How does the procedure work? [INTERVIEWER INSTRUCTIONS:- PROBE: BEFORE, DURING, AFTER]
  - How much does it cost to get circumcised?

- In your opinion is this a reasonable price?

4. PERCEIVED COMMUNITY FEELINGS TO VMMC [15 MIN]

Objective: To explore the role of community influencers in promoting or detracting from the drive to upscale circumcision amongst infants/ adolescents. To uncover the impact of certain community influencers compared to others

- I'm going to name some people in your community and ask you whether they have said anything to you about circumcising baby boys:
  - Pastors/ ministers
  - Community elders
  - Healthcare workers
  - Your partner
  - Your parents
  - Your partner's parents
  - Elders in your family like your grandparents
  - Your friends

[INTERVIEWER INSTRUCTIONS:- IF THE ANSWER IS YES TO ANY OF THE ABOVE, PLEASE ASK WHAT WAS SAID]

- If your pastor/minister in your community advised parents against circumcising their boys but healthcare workers advised parents to circumcising their boys how would people react? Why?

- What would your partner say if you wanted to circumcise your baby boy?

- What would your parents say if you wanted to circumcise your baby boy?

- What would elders in your family, like your grandparents say if you wanted to circumcise your baby boy?

- What do you think your local community would say if you wanted to circumcise your baby boy?

- Of all these people we have spoken about, who do you think would be the biggest supporter of circumcising baby boys?
  - What would they say?
  - Why?
- And who do you think would be most against circumcising baby boys from people who are close to you and people in your community?
  - What would they say?
  - What would make them change their mind?

Of all the people we have spoken about, please put them in order of how important their opinion would be to you if you were considering circumcising your baby boy:

[INTERVIEWER INSTRUCTION: 1 REPRESENTS MOST IMPORTANT AND 8 REPRESENTS LEAST IMPORTANT. RESPONDENT CAN RANK 2 OR MORE PEOPLE AS THE SAME IMPORTANCE IS NECESSARY]

Pastors/ ministers	
Community elders	
Healthcare workers	
Your partner	
Your parents	
Your partner's parents	
Elders in your family like your grandparents	
Friends	

- Why is [# 1] the most important
- Why is [# 8] the least important

5. MAKING THE DECISION TO CIRCUMCISE [10 MIN]

Objective: To understand parental attitudes to circumcising infants vs adolescents vs adult men

- What would people need to know before they could make a decision about whether to circumcise their baby boy or not?
  - Where would they find out this information from?

- Is it better to circumcise baby boys or young/ adolescent boys ?
  - Why?

- What are the risks of circumcising baby boys?
  - Where did you hear about these risks?

- What are the risks of circumcising boys aged 10-14?
  - Where did you hear about these risks?

- Is it better to circumcise baby boys or adult men?
  - Why?

- What would encourage people to actually circumcise their baby boy?

- Do people trust the healthcare facilities to do the best for them and their children?
  - Why? Why not?

- Do people trust their pastors and minister to do the best for them and their children?
  - Why? Why not?

- Do people trust community elders to do the best for them and their children?
  - Why? Why not?
