# Supplementary material for: Attitudes and decision-making about early-infant versus early-adolescent male circumcision: Demand-side insights for sustainable HIV prevention strategies in Zambia and Zimbabwe
Source: PLoS One. 2017 Jul 27;12(7):e0181411. doi: 10.1371/journal.pone.0181411 (PMC5531536; doi:10.1371/journal.pone.0181411)
Supplement: S5 File — (PDF) [file pone.0181411.s006.pdf]

SUSTAINABILITY (S)

© Final Mile Consulting. Confidential document

Scenario

S.Life stage1

Scenario Description

Meet the A's,
They are expectant parents and are making a very important decision regarding the health of their baby. Like most of you they understand the benefits of MC.

The doctor at the clinic is telling the A's that if they have a boy they have the following options regarding MC…..

Hypothesis
At Birth

The first option is to have the baby circumcised at the time of birth. The baby will feel the least pain at that time.

Hypothesis
With Vaccinations

Or they could wait for their baby to be at least a month old and set up an appointment to circumcise the child when they come in for vaccinations.

Hypothesis
Liberal, non-imposing parents

They also have the option to wait till the their son is old enough to make his own decision, even if he decides against it.

Hypothesis
manhood

They could also wait till the their son attains manhood and is sexually active.

Looking at their options…
1. Which one do you think the parents will choose as the best option? and….
2. Which one is not in the best interest of the child?

Remember if your responses is the response of the majority of the people playing the game with you, you stand to make some extra money

You have 30 seconds to answer the question

© Final Mile Consulting. Confidential document

Scenario

S.Advocacy1

Scenario Description

Mr. X is a health worker and is paying a visit to three families today, the A's, the B's , the C's.
Interestingly all the families have recently had a baby boy in the past one month.
Mr. X is trying to register them for MC....

Hypothesis
Self image

In family A the father has signed up to be circumcised and wants his son to do the same

Hypothesis
Care

Where as in family B the mother is very keen on having her baby circumcised because she thinks its good for her child's health and hygiene

Hypothesis
Norm

And lets look at family C where the grand parents think its a good idea to have the child circumcised because circumcision impart of their tradition

So…
Who,do you think Mr. X will be able to register easily?

Remember if your responses is the response of the majority of the people playing the game with you, you stand to make some extra money

You have 30 seconds to answer the question

© Final Mile Consulting. Confidential document

Scenario

S.Advocacy time 2

New

Scenario Description

Meet these four ladies….A, B, C, & D
They all have recently been advised about MC for infants. Each of them came across this information at different times. Lets see…

Hypothesis
at the time of pregnancy

A heard about MC at the time when she was at the clinic to confirm if she was pregnant.

Hypothesis
2 months

B on the other hand is due to deliver in two months and was getting a routine check up when she was informed about MC

Hypothesis
2 days

Then we have C who is due to deliver in two days and was informed about MC at the clinic

Hypothesis
post delivery

Lastly there is D who has just delivered and immediately after the delivery the doctor informed her about MC

Who is most likely to pay heed to the advice and register for MC?

Remember if your responses is the response of the majority of the people playing the game with you, you stand to make some extra money

You have 30 seconds to answer the question

© Final Mile Consulting. Confidential document

Scenario 16

S.Advocacy time 3

Scenario Description

Here is an interesting situation….
Couple A have just gotten their child circumcised. The procedure has just been completed and the family is making their way home.

On the way they bump into another similar family from their community. The A's are talking about how they are feeling having just gone through the procedure….

Hypothesis
Satisfaction

Feeling good about doing something good for their child

Hypothesis
Pride

Having the sense of doing the right thing, looking out for future generations

Hypothesis
Relief

Feeling less anxious and worried about their child contracting infections, HIV, STI's when he grows up

Hmmm…
Which of these feelings do you think got most importance in the conversation?

Remember if your responses is the response of the majority of the people playing the game with you, you stand to make some extra money

You have 30 seconds to answer the question

© Final Mile Consulting. Confidential document

Scenario

S.Motivations1

Scenario Description

Meet the A's,
The are expectant parents and have just found out that they are going to have a baby boy. They are confused if they should or should not get their child circumcised immediately.

They meet three other couples like themselves, the A's, the B's, and the C's, who have gotten their respective sons circumcised, but for different reasons.....

Hypothesis
Hygiene

A's decided that they wanted their son to undergo the procedure because its good hygiene as children don't bathe every day. They think this would reduce infections.

Hypothesis
Prevent pain

B's got the procedure done because its less painful when the boys are young. He may not be able to tolerate the pain when he grows up.

Hypothesis
Protection

Where as C's did it because they were worried that their son may not want to get circumcised when he grows up. We cannot leave such important things for children to decide, they felt.

After listening to these parents, A's have decided to go ahead and get their son circumcised immediately….
Which of the three parents do you think influenced this decision the most?

Remember if your responses is the response of the majority of the people playing the game with you, you stand to make some extra money

You have 30 seconds to answer the question

© Final Mile Consulting. Confidential document

New

Scenario

S.Motivations 3

Scenario Description

Here is Mr. X's wife recently broached the topic MC for their son with him. He is very glad she did so. He just shared this information with four of his friends Mr. A. Mr. B, Mr. C and Mr. D. What impression do the friends get about Mr X's wife ….

Hypothesis
Progressive

A thinks that Mr. X's wife is a progressive mother

Hypothesis
Nurturing

Where as B feels that Mr. X's wife is caring and protecting her child

Hypothesis
Awareness

C's on the other hand feels that Mr X's wife is very aware and knowable woman

Hypothesis
Peer pressure

And then there is D who thinks that Mr. X's wife gets easily influenced by the other ladies

Which friend's impressions is closest to Mr. X's wives real reason for broaching the topic on MC?

Remember if your responses is the response of the majority of the people playing the game with you, you stand to make some extra money

You have 30 seconds to answer the question

© Final Mile Consulting. Confidential document

New

Scenario

S.Mental models 1

Scenario Description

The X's have recently had a baby boy. The young couple wants to get their infant circumcised, but face opposition from the grand parents. They feel that they must respect their elders wishes. They are meeting with four other young parent The A's, B's, and the C's

These couple also faced a similar situation but seem to have over come it….

Hypothesis
out dated

The A's are of the opinion that grand parents will not understand as their time has passed and they are old fashioned

Hypothesis
new context

Where as the B's believe that the grandparents opinion no longer holds value in such health matters as health concerns have changed over the years.

Hypothesis
move on with the times

And lastly the C's feel that If the grand parents had lived in the present times, they'd have done the same as its the prevalent norm

Couple X has actually changed their mind and decided to go ahead with MC
Which couple's rationale, do you think was most influential in changing their mind?

Remember if your responses is the response of the majority of the people playing the game with you, you stand to make some extra money

You have 30 seconds to answer the question

© Final Mile Consulting. Confidential document

New

Scenario

S.Mental models 2

Scenario Description

Meet the A's,
The are expectant parents and have just found out that they are going to have a baby boy. They are confused if they should or should not get their child circumcised immediately.

They meet four other couples like themselves, the A's, the B's, the C's and the D's, who have gotten their respective sons circumcised, but for different reasons.....

Hypothesis
Healing

A's decided that they wanted their son to undergo the procedure because they believed that children heal faster.

Hypothesis
Age and maturity

B's felt that children are not mature enough to make make the right decision so parent need to

Hypothesis
Authority

C's got the procedure done because they felt that their child will thank them later for taking this bold step

Hypothesis
Norms

Where as D's are of the opinion that MC is going to became a norm by the time their child grows up.

After listening to these parents, A's have decided to go ahead and get their son circumcised immediately….
Which of the three parents do you think influenced this decision the most?

Remember if your responses is the response of the majority of the people playing the game with you, you stand to make some extra money

You have 30 seconds to answer the question

© Final Mile Consulting. Confidential document

Scenario

S.Barriers1

Scenario Description

Four young couples in different part of the country recently had baby boys.
All four couples have decided to get their boys circumcised.
A few days before the circumcision they are talking to their neighbours who also have boys.

Hypothesis
Norms

A's neighbours told them that they decided to adhere to the traditional belief that infants between the age of 0-2 months should not be taken out of the house.

Hypothesis
Fear. Anxiety of failed surgery

B's neighbours said they didn't do the procedure as they were anxious that something might go wrong with the procedure and they did not want to risk their child's health

Hypothesis
Bandwagon effect. Anticipation of regret

C's neighbours said they had never heard any other infant in their community undergo the procedure. Didn't want to experiment with their child

Hypothesis
Procrastination. Instant gratification

D's neighbours reasoned that babies are not sexually active. They don't need to undergo the procedure, it can wait.

Among these four families. A, B, C & D, who is most likely drop the idea of MC for their baby?

Remember if your responses is the response of the majority of the people playing the game with you, you stand to make some extra money

You have 30 seconds to answer the question

© Final Mile Consulting. Confidential document

New

Scenario

S.Barriers husband 2

Scenario Description

Here is Mr. X who is a little upset because his wife recently broached the topic MC for their son. He just shared this information with three of his friends Mr. A. Mr. B, and Mr. C.
The friends are trying to guess why the fact that his wife broached the topic of circumcision is bothering Mr. X….

Hypothesis *lack of trust*

A guesses its because Mr. X now feels that his wife is promiscuous and hence she broached the topic

Hypothesis *out cast*

Where as B feels that Mr. X is thinking that his wife is not following community tradition by showing interest in MC

Hypothesis *disrespectful*

C's on the other hand guess that its because Mr. X feels like his wife is not respecting his parents sentiments

So tell us…
Which friend's guess is closest to Mr. X's real reason for being upset with his wife?

Remember if your responses is the response of the majority of the people playing the game with you, you stand to make some extra money

You have 30 seconds to answer the question

© Final Mile Consulting. Confidential document

New

Scenario

S.Barriers3

Scenario Description

Mr. A, B, C, D & E have new born sons. The caters are at their respective health clinics for a regular health check up. They have an opportunity to get their child circumcised now.
Lets see heats going on in their mids…

Hypothesis
changing context

A's thinking that future diseases are going to be different and HIV may not remain a big concern

Hypothesis
out cast

B's thinking that since this is not encouraged in the tribe he would rather not have the son circumcised now

Hypothesis
respectful of an individuals rights

C's thinking that this is a decision that the son should make on his own once he is grown up

Hypothesis
overestimating child's ability

And D's thinking that his son would grow up to be a responsible person and so will not require such measures for preventing HIV

Hypothesis
Anxiety

Where as E is concerned that the child will grow up and blame him for taking such a big decision on his behalf

Who amongst these would be most resistant to advises regarding infant circumcision by the doctors in the hospital?

Remember if your responses is the response of the majority of the people playing the game with you, you stand to make some extra money

You have 30 seconds to answer the question

© Final Mile Consulting. Confidential document

Scenario

S.Barriers 4

Scenario Description

Mrs. A, B, & C have new born sons. They want their son to get circumcised. The nurse in the hospital visits each of these ladies and asks them for their decision regarding the son's circumcision. These women have various different thoughts…

Hypothesis
lack of authority

A's thinking that her decision really doesn't matter. The final decision has to be that of her husband

Hypothesis
Guilt

B's afraid if she pushes for the procedure and if something goes wrong, then she would have to live with the guilt all her life

Hypothesis
Lack of awareness

C's thinking that this is a big decision and that she does not have the knowledge or confidence to make such big decisions

The nurse is now insisting with these mothers to make their decision….
Who would most resist making the decision?

Remember if your responses is the response of the majority of the people playing the game with you, you stand to make some extra money

You have 30 seconds to answer the question

© Final Mile Consulting. Confidential document

Scenario

S.Emotions1

Scenario Description

A and B are a lovely, young couple
Their one month old baby just got circumcised and as they are coming out of the clinic they meet the surgeon. The surgeon asks them how they feel now that their son is circumcised…

The doctor has heard various parents say different things in the past. They include

Hypothesis *Guilt*

They are saying that they feel they should have probably allowed the kid to grow up and make his own decision

Hypothesis *Helplessness*

They are feeling bad that their little one had to go through the pain and they could do little to avoid it

Hypothesis *Relief*

That they have done their bit to protect their son from infections when he grows up

Hypothesis *Anxious*

That they are still anxious if this is enough to protect their son from acquiring infection when he grows up

Hypothesis *Satisfaction*

They have made the absolute right decision and it's something that all parents must do for their kids

1. What do you folks think did this particular couple tell the doctor?
2. Which of these do you think the doctor hears least often?

Remember if your responses is the response of the majority of the people playing the game with you, you stand to make some extra money

You have 30 seconds to answer the question

© Final Mile Consulting. Confidential document

New

Scenario

S.Influencers 1

Scenario Description

Mrs. B is pregnant. And she is aware that the child is a boy. To protect the child from infections in the future she is keen to get her son circumcised soon after his birth. But she is anticipating resistance from the family and community. She is now thinking about the various things that she could do that would reduce the chances of objection at the time when she would want the son to be circumcised.

Hypothesis
Family norm

She is thinking that if she could convince her husband to get circumcised, then there would be little or no objection from her parents and in-laws

Hypothesis
Norm

Or she is thinking that if she could get a tribal leader to speak with her family much in advance, it would help

Hypothesis
Authority

Or is she thinking that if the nurse in the clinic could make strong recommendations for circumcision along with the recommendations they make for the vaccines it would help

Hypothesis
Anticipation of Pain

Or is she thinking that health workers could speak with people in her family and explain to them that the pain is much less when infants are circumcised as compared to when adults get circumcised

A few months later Mrs B's son is born and she manages to get him circumcised without much resistance…
Which of the four actions would you think she chose to reduce resistance to child getting circumcised?

Remember if your responses is the response of the majority of the people playing the game with you, you stand to make some extra money

You have 30 seconds to answer the question

© Final Mile Consulting. Confidential document

New

Scenario

S.Influencers 2

Scenario Description

Mrs. A, B, C, D have new born sons. They have an opportunity to get their child circumcised now. The decision is not made as there are different objections to it.

Hypothesis
Grandparents

A's in a situation where the father wants to get the son circumcised but the grand parents are against the idea

Hypothesis
Tribal leader

B's in a situation where the father and grand parents all want the child to be circumcised, but they know that a leader in their tribe is against the idea of circumcision

Hypothesis
Father

C's situation is that the father does not want to circumcise the son but the grand parents are eager

Hypothesis
Mother

And D herself is not keen to get her son circumcised as she does not want the child to go through such pain at this early stage

Hypothesis
Father

Where as E wants her child circumcised but the father is not for it as he is not circumcised himself

Hmmm…
Who would you think would be least likely to be able to get their son circumcised?

Remember if your responses is the response of the majority of the people playing the game with you, you stand to make some extra money

You have 30 seconds to answer the question

© Final Mile Consulting. Confidential document
