# Supplementary material for: Attitudes and decision-making about early-infant versus early-adolescent male circumcision: Demand-side insights for sustainable HIV prevention strategies in Zambia and Zimbabwe
Source: PLoS One. 2017 Jul 27;12(7):e0181411. doi: 10.1371/journal.pone.0181411 (PMC5531536; doi:10.1371/journal.pone.0181411)
Supplement: S6 File — (PDF) [file pone.0181411.s007.pdf]

Ipsos Healthcare
Minerva House, 5 Montague Close, London SE1 9AY
Tel: + 44 20 3059 5000 Fax: +44 20 3059 4998
Internal client use only

Bill & Melinda Gates Foundation
VMMC Extension - Sustainability
14-026370
Phase 2 : QUANTITATIVE Screener and Questionnaire
Zambia & Zimbabwe

Quotas
(detailed quotas by district for each country are on following pates)

	Zambia	Zimbabwe	TOTALs
Fathers	500	500	1,000
Mothers	500	500	1,000
TOTALs	1,000	1,000	2,000

TEXT COLOR CODING IN QNR:

- **BLUE CAPS = INTERVIEWER NOTES**
- **RED CAPS = PROGRAMMER NOTES**
- **Black text = Questionnaire content**

ZAMBIA QUOTAS

Sustainability Sampling		1,000	50.0%	50.0%	
	**Province**	**District**	**Mothers**	**Fathers**	TOTALs
1	Central	Chibombo	15	15	30
2	Central	Kapiri Mposhi	12	12	24
3	Central	Mumbwa	11	11	22
4	Central	Kabwe	10	10	20
5	Central	Serenje	8	8	16
6	Central	Mkushi	8	8	16
7	Eastern	Chipata	25	25	50
8	Eastern	Kitwe	24	24	48
9	Eastern	Ndola	21	21	42
10	Eastern	Petauke	18	18	36
11	Eastern	Lundazi	17	17	34
12	Eastern	Katete	13	13	26
13	Eastern	Chingola	10	10	20
14	Eastern	Mufulira	7	7	14
15	Eastern	Luanshya	7	7	14
16	Luapula	Mansa	11	11	22
17	Luapula	Samfya	10	10	20
18	Luapula	Nchelenge	8	8	16
19	Luapula	Kawambwa	7	7	14
20	Luapula	Mwense	6	6	12
21	Lusaka	Lusaka	79	79	158
22	Lusaka	Kafue	11	11	22
23	Lusaka	Chongwe	9	9	18
24	Northern	Kasama	14	14	28
25	Northern	Mbala	13	13	26
26	Northern	Mpika	13	13	26
27	Northern	Isoka	10	10	20
28	Northern	Chinsali	9	9	18
29	Northern	Mungwi	8	8	16
30	Northern	Luwingu	8	8	16
31	Northern	Nakonde	7	7	14
32	Northern	Kaputa	7	7	14
33	Northern	Mporokosos	7	7	14
34	Southern	Mazabuka	14	14	28
35	Southern	Kalomo	13	13	26
36	Southern	Choma	13	13	26
37	Southern	Monze	10	10	20
38	Southern	Livingstone	7	7	14
			500	500	1,000

ZIMBABWE QUOTAS

	Sample Sizes by District for Sustainability				
			**Mothers**	**Fathers**	**TOTALs**
1	Bulawayo	BULAWAYO	45	45	**90**
2	Chitungwiza	CHITUNGWIZA	19	19	**38**
3	Harare	HARARE	85	85	**170**
4	Manicaland	MUTARE	22	22	**44**
5	Manicaland	CHIPINGE	14	14	**28**
6	Manicaland	MAKONI	12	12	**24**
7	Manicaland	BUHERA	10	10	**20**
8	Manicaland	MUTASA	9	9	**18**
9	Mashonaland Central	MT. DARWIN	11	11	**22**
10	Mashonaland Central	GURUVE	10	10	**20**
11	Mashonaland Central	BINDURA	9	9	**18**
12	Mashonaland East	GOROMONZI	11	11	**22**
13	Mashonaland East	SEKE	10	10	**20**
14	Mashonaland East	MARONDERA	9	9	**18**
15	Mashonaland East	MUREWA	8	8	**16**
16	Mashonaland West	HURUNGWE	16	16	**32**
17	Mashonaland West	KADOMA	13	13	**26**
18	Mashonaland West	ZVIMBA	12	12	**24**
19	Mashonaland West	MAKONDE	9	9	**18**
20	Masvingo	MASVINGO	16	16	**32**
21	Masvingo	CHIREDZI	13	13	**26**
22	Masvingo	ZAKA	10	10	**20**
23	Masvingo	BIKITA	8	8	**16**
24	Masvingo	CHIVI	8	8	**16**
25	Matabelaland North	HWANGE	8	8	**16**
26	Matabelaland North	BINGA	8	8	**16**
27	Matabelaland North	NKAYI	8	8	**16**
28	Matabeleland South	GWANDA	8	8	**16**
29	Matabeleland South	BEIT BRIDGE	8	8	**16**
30	Matabeleland South	BULILIMA	8	8	**16**
31	Matabeleland South	INSIZA	8	8	**16**
32	Midlands	KWEKWE	17	17	**34**
33	Midlands	GOKWE SOUTH	16	16	**32**
34	Midlands	GWERU	13	13	**26**
35	Midlands	MBERENGWA	9	9	**18**
			**500**	**500**	**1,000**

INTRODUCTION

This survey is being conducted by an independent market research agency on behalf of an international organization.

This research is being conducted to understand attitudes and perceptions about health issues for young boys.

Any information you give will be treated in the strictest confidence and results will only be reported back on an aggregated basis. You will receive an honorarium for your participation.

The following discussion should take about 30 minutes

Are you happy to participate in the interview on this basis?

SINGLE CODE ONLY

1. Yes (CONTINUE)
2. No (CLOSE)

SCREENER

**Section 1 – Screener**		
S0	**SELECT COUNTRY**<br>**ONE ANSWER**	**SINGLE CODE**<br>1. ZAMBIA (CONTINUE)<br>2. ZIMBABWE (CONTINUE)<br>3. OTHER (CLOSE)
S0a	**INTERVIEWER, PLEASE, COMPLETE.**<br>**SETTING.**<br>**ONLY ONE ANSWER**	**SINGLE CODE**<br>1. Urban (CONTINUE)<br>2. Rural (CONTINUE)
S0b	**INTERVIEWER, PLEASE, COMPLETE**<br>**PREFERRED LANGUAGE FOR INTERVIEW.**<br>**ONLY ONE ANSWER**	**SINGLE CODE**<br>1. Bemba (CONTINUE)<br>2. Nyanga (CONTINUE)<br>3. Togna (CONTINUE)<br>4. Shona (CONTINUE)<br>5. Ndebele (CONTINUE)<br>**6.** English (CONTINUE)
S1	**SELECT GENDER OF RESPONDENT**<br>**ONE ANSWER**	**SINGLE CODE**<br>1. Male (CONTINUE)<br>2. Female (CONTINUE)
S2	How many children do you have, not including any unborn children you may be expecting?<br>**ENTER NUMBER**	_ _ # children<br>**RANGE: 0 – 20**<br>**CONTINUE**
S3	**ASK S3 IF S2 > 0; ELSE SKIP T S4**<br>How many of your children are male, if any?<br>**ENTER NUMBER**	_ _ # male children<br>**RANGE: 0 – 20**<br>**IF = 0, CLOSE**
S4	~~**ASK S4 IF S3 > 0; ELSE SKIP TO S5**~~<br>Are any of your male children ages 0 to 2 months old?<br>**SINGLE ANSWER**	**SINGLE CODE**<br>1. Yes (CONTINUE)<br>2. No (CONTINUE)
S5	Are you currently expecting any unborn children from a current pregnancy?<br>**SINGLE ANSWER**	**SINGLE CODE**<br>1. Yes (CONTINUE)<br>2. No (CONTINUE)
S6	**ASK S6 IF S5 = 1 (YES); ELSE SKIP TO S7**<br>Is it possible or probable that the unborn child or children you are expecting may be a boy or boys?<br>**SINGLE ANSWER**	**SINGLE CODE**<br>1. Yes (CONTINUE)<br>2. No (IF S4 = 2 AND S6 = 2, CLOSE)<br>3. Don't know (CONTINUE)
S7	In which province do you currently live?<br>**DO NOT READ OUT LIST**	**USE LIST 1 IN APPENDIX**<br>**SINGLE CODE**
S8	In which district do you currently live?<br>**DO NOT READ OUT LIST**	**USE LIST 2 IN APPENDIX**<br>**SINGLE CODE**

S9	**ASK S9 IF S4 = 1 (YES)**<br>In which province was your youngest boy born?<br>**DO NOT READ OUT LIST**	**USE LIST 1 IN APPENDIX**<br>**SINGLE CODE**
S10	**ASK S10 IF S4 = 1 (YES)**<br>In which district was your youngest boy born?<br>**DO NOT READ OUT LIST**	**USE LIST 2 IN APPENDIX**<br>**SINGLE CODE**
S11	**ASK S11 IF S6 = 1 (YES)**<br>In which province is your possible or probable future son most likely to be born?<br>**DO NOT READ OUT LIST**	**USE LIST 1 IN APPENDIX**<br>**SINGLE CODE**
S12	**ASK S12 IF S6 = 1 (YES)**<br>In which district is your possible or probable future son most likely to be born?<br>**DO NOT READ OUT LIST**	**USE LIST 2 IN APPENDIX**<br>**SINGLE CODE**
S13	**ASK S13 IF S4 = 1 (YES)**<br>Thinking of your boy, aged 0 to 2 months – is he circumcised?	**SINGLE CODE**<br>1. Yes (CLOSE)<br>2. No (CONTINUE)<br>3. Don’t know/Don’t know what circumcision is/unsure (CLOSE)
S14	How old are you?	_ _ years old<br><br>**RANGE: 0-99**<br>**IF < 18, CLOSE**

MAIN QUESTIONNAIRE

Section 2 – Health concerns		
**IF S4 = 1 (YES - INFANT), SHOW THE FOLLOWING TEXT:**<br>As we proceed through the rest of the survey, now, please answer the questions always thinking of your young boy, who is 0 to 2 months old.<br><br>**IF S6 = 1 (YES – UNBORN BOY), SHOW THE FOLLOWING TEXT:**<br>As we proceed through the rest of the survey, now, please answer the questions always thinking of your possible or probable future son.		
A1	When you think about the future for your baby boy, which of the following are the most important to you?<br>*Please select the TWO most important.*<br>**READ LIST**<br>**MUST SELECT TWO FROM LIST**	**MULTICODE**<br>**ALLOW SELECTION OF ONLY TWO ANSWERS;**<br>**MUST SELECT TWO ANSWERS**<br>**RANDOMIZE LIST**<br>1. He is financially successful.<br>2. He is free of diseases.<br>3. He gets a good education.<br>4. He eats well to grow well.<br>5. He becomes someone well recognized in the community.<br>6. He has a family and kids of his own<br>7. He is faithful to his religion
A2	Which of the following are the most important aspects when it comes to your baby's health?<br>*Please select the TWO most important.*<br>**READ LIST**<br>**MUST SELECT TWO FROM LIST**	**MULTICODE**<br>**ALLOW SELECTION OF ONLY TWO ANSWERS;**<br>**MUST SELECT TWO ANSWERS**<br>**RANDOMIZE LIST**<br>1. Getting immunized as soon as possible<br>2. Being protected from HIV infection<br>3. Being breastfed rather than formula<br>4. Being clean and hygienic<br>5. Being protected from future sexually transmitted infections (STIs)<br>6. Being circumcised

Section 3 – HIV knowledge		
B1	Are you aware of any methods for minimizing your own chances of getting infected with HIV?	**SINGLE CODE**<br>1. Yes<br>2. No
B2	**ASK B2 IF B1 = 1 (YES); ELSE SKIP TO SECTION 4**<br>Please tell me ALL the methods you are aware of for minimizing your chances of getting infected with HIV.<br>**DO NOT READ LIST – USE LIST TO CODE ANSWERS**<br>**PROBE UNTIL NO MORE ANSWERS ARE GIVEN – ANY OTHER METHODS YOU ARE AWARE OF?**<br>**SELECT ALL MENTIONED**	**MULTI CODE**<br>1. Using a condom during sex<br>2. Being faithful to one partner<br>3. Abstaining from sex<br>4. ART- Antiretroviral therapy<br>5. Going for HIV counselling at a clinic<br>6. Taking medicine to help me prevent HIV<br>7. If the man is circumcised (VMMC)<br>8. PMTCT- Preventing HIV being transmitted to the baby by the mother<br>9. Other (specify) **– USE POP UP BOX TO CAPTURE**

Section 4 – MC General Knowledge		
C1	Please rate how familiar you feel you are with male circumcision, or VMMC, for adolescent boys or men.<br><br>Rate your feeling of familiarity with it using a 7-point scale where 7 means 'Very Familiar' and 1 means 'Not At All Familiar'. You can use any number from 1 to 7.<br><br>**SHOW SCALE ON DEVICE**<br>**SELECT ONE RATING**	**SINGLE CODE**<br>1. Not At All Familiar<br>2.<br>3.<br>4.<br>5.<br>6.<br>7. Very Familiar
C2	Have any of your male friends or family members been circumcised (to the best of your knowledge)?<br>**SELECT ONLY ONE ANSWER**	**SINGLE CODE**<br>1. Yes<br>2. No
C3	**ASK C3 IF C2=1 (YES, HAVE CIRCUMCISED FRIENDS); ELSE SKIP TO C4**<br>Who of your male friends or family members are circumcised?<br>**DO NOT READ LIST**<br>**PROBE FOR ALL ANSWERS**<br>**SELECT ALL MENTIONED**	**MULTI CODE**<br>1. Father<br>2. Brother(s)<br>3. Cousin(s)<br>4. Uncle(s)<br>5. Grandfather<br>6. One of your friends<br>7. Several friends<br>8. Others /SPECIFY/ - **USE POP-UP BOX TO CAPTURE**
C4	**ASK C4 IF C1 = RATING OF 2-7; ELSE, IF C1 = 1, SKIP TO C6**<br>What, if anything, BAD have you heard about male circumcision, or VMMC?<br>**PROBE: WHAT ELSE?**<br>**CAPTURE ANSWERS IN DETAIL IN ENGLISH**	**OPEN END**
C5	**ASK C5 IF C1 = RATING OF 2-7; ELSE, IF C1 = 1, SKIP TO C6**<br>What, if anything, GOOD have you heard about male circumcision, or VMMC?<br>**PROBE: WHAT ELSE?**<br>**CAPTURE ANSWERS IN DETAIL IN ENGLISH**	**OPEN END**
C6	Which of the following statements BEST reflects what you think about a circumcised man being able to get infected with HIV?<br>**READ LIST**<br>**SELECT ONE**	**SINGLE CODE**<br>1. Circumcision completely protects a man from getting HIV from his partner<br>2. Circumcision partially protects a man from getting HIV from his partner<br>3. Circumcision does not affect a man's chances of getting HIV from his partner.<br>4. Circumcision increases the chances of getting HIV from his partner<br>5. Unsure about how circumcision affects the risks of getting HIV.

C7	To what extent do you agree with each of the following statements about male circumcision?<br><br>Please indicate how much you agree or disagree with each statement using a 7-point scale, where 1 means 'Strongly Disagree', 4 means 'Neither Agree Nor Disagree' and 7 means 'Strongly Agree'.<br>**READ AND SHOW SCALE**<br>**SELECT ONE RATING FOR EACH STATEMENT**<br>**REPEAT SCALE IF NECESSARY**	**SINGLE RATING FOR EACH STATEMENT**<br>1. Strongly Disagree<br>2.<br>3.<br>4. Neither Agree Nor Disagree<br>5.<br>6.<br>7. Strongly Agree<br><br>**RANDOMISE STATEMENTS**<br>1. Male circumcision is completely safe<br>2. Male circumcision is for every boy or man<br>3. Going for circumcision is embarrassing<br>4. Male circumcision improves personal hygiene by making the penis cleaner<br>5. Being circumcised will lower a man's risk of getting infected with STIs and HIV<br>6. Male circumcision will protect a man's female partner from HPV<br>7. A man being circumcised makes it easier for him to have his sons circumcised<br>8. The procedure for male circumcision is very painful<br>9. The healing process for male circumcision is very painful<br>10. Women prefer circumcised men<br>11. It is possible to circumcise boys at school<br>12. It is possible to circumcise baby boys within their first 60 days after birth<br>13. Male circumcision is becoming common and it is going to become the norm in society<br>14. Men who are circumcised are role models for the community<br>15. I trust the healthcare professionals who perform male circumcision<br>16. The benefits from male circumcision are worth the risks<br>17. The healing time after getting circumcised is too long<br>18. It's concerning what will be done with the foreskin after it is removed<br>19. I don't know where to go for circumcision<br>20. Male circumcision can cause fatal bleeding<br>21. Male circumcision can cause complications during the healing process (for example, infections)<br>22. The penis would be less sensitive during sex after being circumcised<br>23. Male circumcision can cause loss of potency<br>24. Male circumcision is inappropriate because you shouldn't change the way God created a boy or man<br>25. Male circumcision is only for people who belong to particular tribes or social groups

		26. Circumcised men will be more promiscuous - they will sleep around more<br>27. Many men in my community have already been circumcised<br>28. Many men I know have already been circumcised<br>29. Men who are circumcised have better sexual relationships with their partners<br>30. Men in monogamous relationships do not need to get circumcised<br>31. Circumcision is not worth it since a man still needs to wear a condom
C8	To what degree would you trust each of the following sources of information about male circumcision?<br><br>Please indicate how much you trust each using a 7-point scale, where 1 means 'Do Not Trust At All' and 7 means 'Completely Trust'.<br>**READ AND SHOW SCALE**<br>**SELECT ONE RATING FOR EACH SOURCE**<br>**REPEAT SCALE IF NECESSARY**	**SINGLE RATING FOR EACH STATEMENT**<br>1. Do Not Trust At All<br>2.<br>3.<br>4.<br>5.<br>6.<br>7. Completely Trust<br><br>**RANDOMISE SOURCES**<br>1. Your spouse or partner<br>2. Your son<br>3. Your brother<br>4. Your father<br>5. Your grandfather<br>6. Your mother<br>7. Your grandmother<br><br>9. Male friend<br>10. Female friend<br>11. Teacher<br>12. Community mobilizer<br>13. Doctor, nurse or healthcare worker<br>14. Celebrity<br>15. Chief or other community leader<br>16. Church leader or other religious leader<br>17. Posters / Billboards / Signs<br>18. Radio<br>19. Television / TV<br>20. Newspapers or magazines<br>21. Internet
C9	What is the most common age at which men in your community tend to get circumcised, medically or traditionally, if they do at all?<br>**READ LIST**<br>**SELECT ONE**	**SINGLE CODE**<br>1. 0 to 2 months after birth<br>2. Ages 10-13 years<br>3. Ages 14-17 years<br>4. Ages 18-25 years<br>5. Ages 25 years or older<br>6. Never<br>7. Unsure

C10	What do you think is the ideal age for a boy or man to be medically circumcised?<br>**READ LIST**<br>**SELECT ONE**	**SINGLE CODE**<br>1. 0 to 2 months after birth<br>2. Ages 10-13 years<br>3. Ages 14-17 years<br>4. Ages 18-25 years<br>5. Ages 25 years or older<br>6. Never<br>7. Unsure
C11	Why do you think this is the ideal age for a boy or man to be medically circumcised?<br>**CAPTURE ANSWERS IN DETAIL IN ENGLISH**	**OPEN END**

Section 5 – EIMC knowledge		
D1	Have you previously heard about circumcising baby boys, ages 0 to 2 months after birth?<br>**SELECT ONE**	**SINGLE CODE**<br>1. Yes<br>2. No
D2	**ASK D2 IF D1 = 1 (YES); ELSE SKIP TO D4**<br>From which sources have you previously heard about circumcising baby boys, ages 0 to 2 months after birth?<br>**DO NOT READ LIST**<br>**PROBE FOR ALL ANSWERS**<br>**SELECT ALL MENTIONED**	**MULTICODE**<br>1. Your spouse or partner<br>2. Your son<br>3. Your brother<br>4. Your father<br>5. Your grandfather<br>6. Your mother<br>7. Your grandmother<br>8. Other family member<br>9. Male friend<br>10. Female friend<br>11. Teacher<br>12. Community mobilizer<br>13. Doctor, nurse or healthcare worker<br>14. Celebrity<br>15. Chief or other community leader<br>16. Church leader or other religious leader<br>17. Posters / Billboards / Signs<br>18. Radio<br>19. Television / TV<br>20. Newspapers or magazines<br>21. Internet<br>22. Other – specify - **USE POP-UP BOX TO CAPTURE**<br>23. Cannot remember / cannot say

D3	**ASK D3 IF AT LEAST ONE SOURCE (1-22) SELECTED IN D2; ELSE SKIP TO D4**<br>How useful was the information you received from each source about circumcising baby boys, ages 0 to 2 months after birth?<br><br>Please indicate how useful the information was from each source, using a 7-point scale, where 1 means 'Not At All Useful' and 7 means 'Extremely Useful'.<br>**READ AND SHOW SCALE**<br>**SELECT ONE RATING FOR EACH SOURCE SHOWN**<br>**REPEAT SCALE IF NECESSARY**	**SINGLE RATING FOR EACH SOURCE**<br>1. Not At All Useful<br>2.<br>3.<br>4.<br>5.<br>6.<br>7. Extremely Useful<br><br>**ONLY SHOW SOURCES SELECTED IN D2**<br>1. Your spouse or partner<br>2. Your son<br>3. Your brother<br>4. Your father<br>5. Your grandfather<br>6. Your mother<br>7. Your grandmother<br>8. Other family member<br>9. Male friend<br>10. Female friend<br>11. Teacher<br>12. Community mobilizer<br>13. Doctor, nurse or healthcare worker<br>14. Celebrity<br>15. Chief or other community leader<br>16. Church leader or other religious leader<br>17. Posters / Billboards / Signs<br>18. Radio<br>19. Television / TV<br>20. Newspapers or magazines<br>21. Internet<br>22. Other source

D4	To what degree would you trust each of the following sources of information about circumcising baby boys, ages 0 to 2 months after birth?<br><br>Please indicate how much you trust each using a 7-point scale, where 1 means 'Do Not Trust At All' and 7 means 'Completely Trust'.<br>**READ AND SHOW SCALE**<br>**SELECT ONE RATING FOR EACH SOURCE**<br>**REPEAT SCALE IF NECESSARY**	**SINGLE RATING FOR EACH SOURCE**<br>1. Do Not Trust At All<br>2.<br>3.<br>4.<br>5.<br>6.<br>7. Completely Trust<br><br>**RANDOMISE SOURCES**<br>1. Your spouse or partner<br>2. Your son<br>3. Your brother<br>4. Your father<br>5. Your grandfather<br>6. Your mother<br>7. Your grandmother<br><br>9. Male friend<br>10. Female friend<br>11. Teacher<br>12. Community mobilizer<br>13. Doctor, nurse or healthcare worker<br>14. Celebrity<br>15. Chief or other community leader<br>16. Church leader or other religious leader<br>17. Posters / Billboards / Signs<br>18. Radio<br>19. Television / TV<br>20. Newspapers or magazines<br>21. Internet
D5	**ASK D5 IF D1 = 1 (YES)**<br>Do people in your community circumcise their infants?<br>**SELECT ONE**	**SINGLE CODE**<br>1. Yes<br>2. No

Section 6 – EIMC attitudes (drivers & barriers)		
**SHOW THE FOLLOWING TEXT:**<br>In the next set of questions we'll talk about early infant circumcision, which is the circumcision of baby boys during the age of 0 to 2 months old.		
E1	In your opinion, how acceptable to people in your community would be circumcising baby boys at the age of 0 to 2 months old?<br><br>Please rate your view of the acceptability using the following 7-point scale where 1 means 'Not At All Acceptable' and 7 means 'Completely Acceptable'.<br>**SHOW SCALE ON DEVICE**<br>**SINGLE RATING**	**SINGLE CODE**<br>1. Not At All Acceptable<br>2.<br>3.<br>4.<br>5.<br>6.<br>7. Completely Acceptable
E2	Do you personally know anyone who has circumcised their baby boy, aged 0 to 2 months old?<br>**SELECT ONE**	**SINGLE CODE**<br>1. Yes<br>2. No
E3	How likely would you be to circumcise your baby boy when he is 0 to 2 months old, if the procedure was free of cost?<br><br>Please answer using the following scale…<br>**READ AND SHOW SCALE**<br>**SELECT ONE RATING**	**SINGLE CODE**<br>1. Definitely would not<br>2. Probably would not<br>3. May or may not<br>4. Probably would<br>5. Definitely would
E4	**ASK E4 IF E3 = 2-5; ELSE SKIP TO E6**<br>In which of the following settings would you consider having your infant boy get circumcised, when he is 0 to 2 months old?<br>**READ LIST**<br>**SELECT ALL THAT APPLY**	**MULTICODE**<br>1. General hospital<br>2. Local clinic<br>3. Mobile services<br>4. Home<br>5. Traditional ceremony<br>6. A center which specializes in circumcision<br>**7.** Other place (specify) – **USE POP UP TO CAPTURE**<br>8. Unsure/I don't know – **SINGLE CODE**

E5	**ASK E5 IF E3 = 2-5; ELSE SKIP TO E6**<br>How likely would you be to circumcise your baby boy when he is 0 to 2 months old, if you had to pay **<INSERT AMOUNT FROM LIST >** to have the procedure done?<br>**READ AND SHOW SCALE**<br>**SELECT ONE RATING**<br><br>**REPEAT QUESTION FOR EACH COST LEVEL, STARTING AT LOWEST AND MOVING HIGHER UNTIL LIST IS COMPLETE (6 COST LEVELS)**	**SINGLE CODE FOR EACH COST LEVEL**<br>1. Definitely would not<br>2. Probably would not<br>3. May or may not<br>4. Probably would<br>5. Definitely would<br><br>**ZAMBIA COSTS (USE THIS LIST IF S0 = 1):**<br>1. 7.50 Kwacha<br>2. 20.00 Kwacha<br>3. 35.00 Kwacha<br>4. 55.00 Kwacha<br>5. 75.00 Kwacha<br>6. 110.00 Kwacha<br><br>**ZIMBABWE COSTS (USE THIS LIST IF S0 = 2):**<br>1. $ 1.00 USD<br>2. $ 2.50 USD<br>3. $ 5.00 USD<br>4. $ 7.50 USD<br>5. $ 10.00 USD<br>6. $ 15.00 USD
E6	To what extent do you agree with each of the following statements about the influences for you to decide to circumcise your baby boy while he is 0 to 2 months old?<br><br>Please indicate how much you agree or disagree with each statement using a 7-point scale, where 1 means 'Strongly Disagree', 4 means 'Neither Agree Nor Disagree and 7 means 'Strongly Agree'.<br>**READ AND SHOW SCALE**<br>**SELECT ONE RATING FOR EACH STATEMENT**<br>**REPEAT SCALE IF NECESSARY**	**SINGLE RATING FOR EACH STATEMENT**<br>1. Strongly Disagree<br>2.<br>3.<br>4. Neither Agree Nor Disagree<br>5.<br>6.<br>7. Strongly Agree<br><br>**RANDOMISE STATEMENTS**<br>1. I would circumcise my baby boy, while he is 0 to 2 months old, even if my spouse/partner was against it.<br>2. I would circumcise my baby boy, while he is 0 to 2 months old, even if my parents were against it.<br>3. I would circumcise my baby boy, while he is 0 to 2 months old, even if my spouse's/partner's parents were against it.<br>4. My religious beliefs would strongly influence my decision on whether to circumcise my baby boy, while he is 0 to 2 months old.<br>5. I would circumcise my baby boy, while he is 0 to 2 months old, because it is becoming the social norm and most boys and men will be circumcised in the future<br>6. The protection from risk of HIV infection for my son would strongly influence me toward deciding to circumcise my baby

		boy while he is 0 to 2 months old.<br>7. The hygiene benefits for his penis would strongly influence me toward deciding to circumcise my baby boy, while he is 0 to 2 months old.<br>8. Improved sexual performance for my son when he is older would strongly influence me toward deciding to circumcise my baby boy while he is 0 to 2 months old.
E7	What are the reasons you would NOT consider circumcising your baby boy while he is 0 to 2 months old?<br>**PROBE FULLY FOR ALL REASONS – ASK, AND WHAT OTHER REASONS?**<br>**CAPTURE ANSWERS IN DETAIL IN ENGLISH**	**OPEN END**
E8	To what extent do you agree with each of the following statements about the influences for you to decide NOT to circumcise your baby boy while he is 0 to 2 months old?<br><br>Please indicate how much you agree or disagree with each statement using a 7-point scale, where 1 means 'Strongly Disagree', 4 means 'Neither Agree Nor Disagree and 7 means 'Strongly Agree'.<br>**READ AND SHOW SCALE**<br>**SELECT ONE RATING FOR EACH STATEMENT**<br>**REPEAT SCALE IF NECESSARY**	**SINGLE RATING FOR EACH STATEMENT**<br>1. Strongly Disagree<br>2.<br>3.<br>4. Neither Agree Nor Disagree<br>5.<br>6.<br>7. Strongly Agree<br><br>**RANDOMISE STATEMENTS**<br>1. I would NOT circumcise my baby boy, while he is 0 to 2 months old, even if my spouse/partner wanted it.<br>2. I would NOT circumcise my baby boy, while he is 0 to 2 months old, even if my parents wanted it.<br>3. I would NOT circumcise my baby boy, while he is 0 to 2 months old, even if my spouse's/partner's parents wanted it.<br>4. Circumcising my baby boy when he is 0 to 2 months old is against my religious beliefs<br>5. 0 to 2 months is too young to circumcise a boy<br>6. I would not want my baby to go through pain the pain of circumcision at the age of 0 to 2 months<br>7. It should be my son's own decision when he grows up whether he gets circumcised or not.<br>8. I don't trust the people carrying out the procedure to have my son circumcised at the age of 0 to 2 months.<br>9. The benefits do NOT outweigh the risks for me to circumcise my baby boy while he is 0 to 2 months old.<br>10. I worry about a bad outcome if I get my baby boy circumcised while he is 0 to 2 months old.

		11. It would be too expensive for me to get my baby boy circumcised while he is 0 to 2 months old.
E9	What benefits, if any, can you think of for circumcising your son while he is 0 to 2 months old?<br>**PROBE UNTIL NO MORE ANSWERS ARE GIVEN – ANY OTHER BENEFITS YOU CAN THINK OF? CAPTURE ANSWERS IN DETAIL IN ENGLISH**	**OPEN END**
E10	Which of the following would you consider the three greatest benefits for getting your son circumcised while he is 0 to 2 months old?<br><br>Select up to 3 of these benefits for early infant male circumcision.<br><br>**READ LIST**<br>**SELECT UP TO 3 FROM LIST**<br>**CAN SELECT JUST 1 OR 2, IF RESPONDENT DOES NOT IDENTIFY 3**	**MULTICODE. ALLOW TO SELECT 3 OPTIONS**<br>**RANDOMIZE LIST**<br>1. HIV protection: my son will have less chances of contracting HIV<br>2. My son won't remember the pain<br>3. Less pain: my son will fell less pain than when he is an adult<br>4. Faster healing; the wound heals quicker for infants<br>5. Lower risk of infection: I can monitor the wound and take care of it better<br>6. Hygiene benefits: my son will grow up with a clean penis<br>7. No difficult decision: my son won't have to struggle with the decision to get circumcised – it's already done when he's older<br>8. Sexual benefits: my son will have a better sexual life<br>9. No abstinence: my son will not have to abstain from sex during the healing time for circumcision when he is older<br>10. None of these

E11	What concerns, if any, do you have for circumcising your son while he is 0 to 2 months old?<br>**PROBE UNTIL NO MORE ANSWERS ARE GIVEN – ANY OTHER CONCERNS YOU HAVE?**<br>**CAPTURE ANSWERS IN DETAIL IN ENGLISH**	**OPEN END**
E12	Who would play the MOST important role in deciding whether your baby boy would be circumcised, while he is 0 to 2 months old?<br>**SELECT ONE**	**SINGLE CODE**<br>1. Baby's mother<br>2. Baby's father<br>3. Mother's partner at the time of circumcision (if he's not the baby father)<br>4. The father's father<br>5. The father's mother<br>6. Other male family of the father (brothers, uncles)<br>7. Other female family of the father (sisters, aunties)<br>8. The mother's father<br>9. The mother's mother<br>10. Other male family of the mother (brothers, uncles)<br>11. Other female family of the mother (sisters, aunties)<br>12. Brothers or sisters of the baby<br>13. Religious leader<br>14. Traditional leader<br>15. Traditional healer<br>16. Doctor, nurses and other healthcare professionals<br>17. Other – specify – **USE POP UP TO CAPTURE**

E12	Who else would play the <u>important roles</u> in deciding whether your baby boy would be circumcised, while he is 0 to 2 months old?<br>**PROBE – ANY OTHERS?**<br>**SELECT ALL MENTIONED**	**MULTI CODE**<br>**EXCLUDE SELECTION MADE IN E11 FROM LIST BELOW**<br>1. Baby's mother<br>2. Baby's father<br>3. Mother's partner at the time of circumcision (if he's not the baby father)<br>4. The father's father<br>5. The father's mother<br>6. Other male family of the father (brothers, uncles)<br>7. Other female family of the father (sisters, aunties)<br>8. The mother's father<br>9. The mother's mother<br>10. Other male family of the mother (brothers, uncles)<br>11. Other female family of the mother (sisters, aunties)<br>12. Brothers or sisters of the baby<br>13. Religious leader<br>14. Traditional leader<br>15. Traditional healer<br>16. Doctor, nurses and other healthcare professionals<br>17. Other – specify – **USE POP UP TO CAPTURE**
E13	If you strongly wanted your baby to be circumcised while he is 0 to 2 months old, would his other parent need to agree also, or could you make the decision alone?	**SINGLE CODE**<br>1. Yes, my son's other parent would have to agree<br>2. No, I can make this decision without consulting my son's other parent<br>3. Unsure/I don't know
E14	To what extent do you agree with the each of the following statements about infant, adolescent and adult male circumcision?<br><br>Please indicate how much you agree or disagree with each statement using a 7-point scale, where 1 means 'Strongly Disagree', 4 means 'Neither Agree Nor Disagree and 7 means 'Strongly Agree'.<br>**READ AND SHOW SCALE**<br>**SELECT ONE RATING FOR EACH STATEMENT**<br>**REPEAT SCALE IF NECESSARY**	**SINGLE RATING FOR EACH STATEMENT**<br>1. Strongly Disagree<br>2.<br>3.<br>4. Neither Agree Nor Disagree<br>5.<br>6.<br>7. Strongly Agree<br><br>**RANDOMISE STATEMENTS**<br>1. A baby's circumcision wound will heal faster than an adult's circumcision wound.<br>2. A baby's circumcision wound will heal quicker than an adolescent's circumcision wound.<br>3. A baby will suffer less pain during and after circumcision than an adult will suffer<br>4. A baby will suffer less pain during and after circumcision than an adolescent will

		suffer<br>5. By the time my son grows up, circumcision will be the norm.<br>6. Circumcision could be used to discriminate among tribes and communities.<br>7. I wouldn't get my baby circumcised because I don't know anyone who has done it yet.<br>8. It is better to let the child decide about whether to get circumcised or not.<br>9. I trust the healthcare professionals that practice circumcision<br>10. I believe the facilities in our hospitals are appropriate for circumcision procedures.<br>11. Most men will be circumcised when my son is older and he will be happy that I made this decision for him<br>12. Circumcision will protect my son from future sexual diseases<br>13. It will be easier to keep his penis clean and prevent infections while my son is a baby and young boy<br>14. I will be able to care for his wound more easily while he is a baby, which means that healing will be easier<br>15. Circumcision is safer for a baby than for an older boy or man<br>16. It is less painful for a baby to be circumcised than for an older boy or man<br>17. I will feel relief that I have done something good for the future of my son<br>18. I am concerned about what will happen to my son's foreskin after the procedure<br>19. If my child were to get HIV in the future, I would regret not circumcising him as an infant<br>20. Many men wish that they had been circumcised as infants or adolescents, so they wouldn't have to face doing it as an adult<br>21. It is important to include traditional circumcision ceremonies along with the medical procedure
E15	When is the best time for a healthcare worker to tell parents about the option to circumcise their son as an infant, while he is 0 to 2 months old?<br>**SELECT ONE**	**SINGLE CODE**<br>1. Before a couple is pregnant<br>2. When a couple first finds out that they are pregnant<br>3. During the pregnancy, before the baby is born<br>4. Immediately after the baby boy is born<br>5. Some weeks after the baby is born<br>6. Never

Section 7> Personal Information		
SHOW THE FOLLOWING TEXT<br>The following questions may contain sensitive information. Remember that all your responses will be confidential and remain anonymous.		
F1	IF S1 = 1 (MALE), SHOW THIS TEXT<br>Are you circumcised?<br>IF S1 = 2 (FEMALE), SHOW THIS TEXT<br>Is your partner circumcised?<br>SELECT ONE	SINGLE CODE<br>1. Yes, circumcised<br>2. No, not circumcised<br>3. I don't know<br>4. Prefer not to answer
F2	Do you know anyone close to you who is infected with HIV?<br>SELECT ONE	SINGLE CODE<br>1. Yes, I know someone<br>2. No, I don't know anyone<br>3. Prefer not to answer
F3	Are you aware of your own HIV status?<br>SELECT ONE	SINGLE CODE<br>1. Yes<br>2. No<br>3. Not sure<br>4. Prefer not to answer
F4	ASK F4 IF F3 = 1 (YES); ELSE SKIP TO F6<br>May I ask you your HIV status?<br>SELECT ONE	SINGLE CODE<br>1. Yes<br>2. No
F5	ASK F5 IF F4 = 1 (YES); ELSE SKIP TO F6<br>Are you HIV POSITIVE?<br>SELECT ONE	SINGLE CODE<br>1. Yes<br>2. No<br>3. Prefer not to answer
F6	IF S1 = 1 (MALE), SHOW THIS TEXT<br>Is the mother of the baby HIV infected?<br>IF S1 = 2 (FEMALE), SHOW THIS TEXT<br>Is the father of the baby HIV infected?<br>SELECT ONE	SINGLE CODE<br>1. Yes<br>2. No<br>3. I don't know<br>4. Prefer not to answer

SECTION 8: DEMOGRAPHICS

ASK ALL RESPONDENTS

R1. From which tribe is your father's side of your family?

DO NOT READ LIST

ONE ANSWER

	**PROGR: SHOW THE FOLLOWING LIST IF S1=1 (ZAMBIA)**
1	Bemba
2	Bisa
3	Chewa
4	Kaonde
5	Lala
6	Lamba
7	Lenje
8	Lozi
9	Lunda
10	Luvale
11	Mambwe
12	Mbunda
13	Namwanga
14	Ngoni
15	Nsenga
16	Tonga
17	Tumbuka
18	Ushi
99	Other tribe (specify) **– USE POP-UP TO CAPTURE**

	**PROGR: SHOW THE FOLLOWING LIST IF S1=2 (ZIMBABWE)**
1	Ndebele
2	Zezuru
3	Karanga
4	Manyika
5	Ndawu
6	Korekore
7	Kalanga
8	Xhosa
9	Sotho
10	Nyanja
11	Tonga
12	Tumbuka
13	Venda
99	Other tribe (specify) **– USE POP-UP TO CAPTURE**

ASK ALL RESPONDENTS

R2. From which tribe is your mother's side of your family?

DO NOT READ LIST

ONE ANSWER

	**PROGR: SHOW THE FOLLOWING LIST IF S1=1 (ZAMBIA)**
1	Bemba
2	Bisa
3	Chewa
4	Kaonde
5	Lala
6	Lamba
7	Lenje
8	Lozi
9	Lunda
10	Luvale
11	Mambwe
12	Mbunda
13	Namwanga
14	Ngoni
15	Nsenga
16	Tonga
17	Tumbuka
18	Ushi
99	Other tribe (specify) – **USE POP-UP TO CAPTURE**

	**PROGR: SHOW THE FOLLOWING LIST IF S1=2 (ZIMBABWE)**
1	Ndebele
2	Zezuru
3	Karanga
4	Manyika
5	Ndawu
6	Korekore
7	Kalanga
8	Xhosa
9	Sotho
10	Nyanja
11	Tonga
12	Tumbuka
13	Venda
99	Other tribe (specify) – **USE POP-UP TO CAPTURE**

ASK ALL RESPONDENTS

R3. How would you classify your religion?

DO NOT READ LIST

ONE ANSWER

01	Traditional
02	Presbyterianism
03	Roman Catholic
04	Anglican
05	Methodist
06	Pentecostal
07	New Apostolic Church
08	Lutheran
09	Seventh-day Adventist
10	Muslim
11	Other /SPECIFY/___________ **– USE POP-UP TO CAPTURE**
99	None

ASK ALL RESPONDENTS

R4. Which of the following best describes your working status?

READ LIST

ONE ANSWER

1	Employed
2	Unemployed (but seeking work)
3	Unemployed (and NOT seeking work)
5	Student
99	Other (describe) **– USE POP-UP TO CAPTURE**

ASK ALL RESPONDENTS

R5. What is the highest level of education you have completed? (Single answer)

READ LIST. ONE ANSWER

	**PROGR: SHOW THE FOLLOWING LIST IF S1=1 (ZAMBIA)**
1	None
2	Some primary(1-8 years)<br>(Up to Grade 7 / Standard 5)
3	Completed primary (8 years)<br>(Up to Grade 7 / Standard 5)
4	Some secondary (9-12)<br>(Grade 8 – Grade 12 / Standard 6 – Matric)
5	Completed secondary (12 years)<br>(Grade 12 / Matric completed)
6	Some university<br>(Including Technikon diplomas/degrees)
7	Completed undergraduate university degree<br>(Including Technikon diplomas/degrees)
8	Some graduate university<br>(Honours degree obtained)
9	Completed graduate degree<br>(Honours degree obtained)
10	Some post-graduate university<br>(Masters/Ph.d etc.)
11	Completed post-graduate degree<br>(Masters/Ph.d etc.)

	**PROGR: SHOW THE FOLLOWING LIST IF S1=2 (ZIMBABWE)**
1	None
2	Some primary(1-7 years)<br>(Up to Grade 7 /)
3	Completed primary (7years)<br>(Up to Grade7)
4	Some secondary<br>(Form 1-4/)
5	Completed secondary<br>(Ordinary Level)
6	Completed secondary<br>(Advanced Level)
7	Some Tertiary Education<br>(diplomas)
8	Completed Tertiary Education<br>(diplomas)
9	Some graduate university<br>(Honours degree obtained)
10	Completed graduate degree<br>(Honours degree obtained)
11	Some post-graduate university<br>(Masters/Ph.d etc.)

12	Completed post-graduate degree (Masters/Ph.d etc.)

ASK R6 IF S5 >=18 (18+ YEARS OLD)

R6. What is your relationship Status? Please give one answer only.

READ LIST

MULTIPLE ANSWERS POSSIBLE FOR THE FIRST 3 ANSWERS OTHERWISE ONE ANSWER

ALLOW CODES 1, 2 AND 3 TO ALL BE SELECCTED AS MULTIPLE ANSWERS	
1	Married – traditionally
2	Married – legally
3	Married – church
4	Single and no partner
5	Single but have a girlfriend/Partner
6	Single but have multiple girlfriends/Partners
7	Separated and no partner
8	Separated but have a girlfriend/partner
9	Separated but have multiple girlfriends/partners
10	Married and have a girlfriend/partner (other than wife)
11	Married but have multiple girlfriends/other partners
12	Widower and have no partner
13	Widower, but have a girlfriend/partner
14	Widower, but have multiple girlfriends/partners

ASK R7 IF R6 = 1-3, 5-6, 8-11 OR 13-14

R7. Do you live with your partner(s)?

ONE ANSWER

Yes	1
No	2

ASK ALL RESPONDENTS

R8. I am going to read out a list of things. Some of these things might sound strange to you, but we need to ask them to understand a bit more about your lifestyle.
Answer Yes or No for each of the following questions.

		YES	NO	SCORE TO ADD IF YES
1	Do you have a color TV?			+18
2	Did you access the Internet during the past 4 weeks?			+48
3	Do you have a satellite dish/DSTV/cable TV subscription?			+34
4	Do you have a built-in kitchen sink in your kitchen?			+31
5	Do you have a microwave oven?			+32
6	Did you read a newspaper in the last 7 days?			+17
7	Do you have a video recorder?			+18
8	Do you have a cell phone/mobile phone with a working line?			+16
9	Do you have an electric iron?			+17
10	Do you have a personal computer for your own personal use at home?			+34
11	Do you have a fixed telephone line at home or an outstanding application for one?			+14
12	Did you watch TV in the last 7 days?			+17
13	Do you have access to e-mail?			+41
14	Do you have an automatic washing machine?			+32
15	Do you have refrigerator?			+20
16	Do you have a hi-fi or music center?			+17
17	Do you have a free-standing deep freezer?			+19
18	Do you have a video camera/camcorder?			+35
19	Do you have an account with a commercial bank?			+15
20	Do you live in a house, cluster house or condominium?			+11
21	How many cars do you have in your household? One answer only.			
22	None			+0
23	One			+12
24	Two or more			+24
25	Did you buy adult clothing in the past six months?			+10
**26**	**ADD THIS EVERY TIME (CONSTANT)**			+32

R9 – CREATE VARIABLE = SUM OF SCORES FROM YES ANSWERS IN R8

R10 – CREATE VARIABLE = CODED AS FOLLOWS:

**1**	**SCORE = 319 – 999**	**LSM 13 – 17 (AB)**
**2**	**SCORE = 187 – 318**	**LSM 9 – 12 (C1)**
**3**	**SCORE = 88 – 186**	**LSM 5 – 8 (C2)**
**4**	**SCORE = 0 – 87**	**LSM 1 – 4 (D)**

Those are all the questions I have. Thank you so much for your participation in this research.

<END>

Appendix

LIST 1

Central	1	USE THE FOLLOWING LIST IF S0 = 1 (ZAMBIA)
Copperbelt	2	
Eastern	3	
Luapula	4	
Lusaka	5	
North Western	6	
Muchinga	7	
Northern	8	
Southern	9	
Western	10	
Bulawayo	11	USE THE FOLLOWING LIST IF S0 = 2 (ZIMBABWE)
Chitungwiza	12	
Harare	13	
Manicaland	14	
Mashonaland Central	15	
Mashonaland East	16	
Mashonaland West	17	
Masvingo	18	
Matabelaland North	19	
Matabeleland South	20	
Midlands	21	

LIST 2

1	CHIBOMBO	SHOW IF S0 = 1 (ZAMBIA) AND LIST 1 = 1 (CENTRAL)
2	KAPIRI MPOSHI	
3	MUMBWA	
4	KABWE	
5	SERENJE	
6	MKUSHI	
7	KITWE	SHOW IF S0 = 1 (ZAMBIA) AND LIST 1 = 1 (COPPERBELT)
8	NDOLA	
9	CHINGOLA	
10	CMUFULIRA	
11	LUANSHYA	
12	CHILILABOMBWE	
13	MPONGWE	
14	MASAITI	
15	KALULUSHI	
16	LUFWANYAMA	
17	CHIPATA	SHOW IF S0 = 1 (ZAMBIA) AND LIST 1 = 1 (EASTERN)
18	PETAUKE	
19	LUNDAZI	

20	KATETE	
21	ECHADIZA	
22	CHAMA	
23	NYIMBA	
24	MAMBWE	
25	MANSA	**SHOW IF S0 = 1 (ZAMBIA) AND LIST 1 = 1 (LUAPULA)**
26	SAMFYA	
27	NCHELENGE	
28	KAWAMBWA	
29	MWENSE	
30	CHIENGI	
31	MILENGI	
32	LUSAKA	**SHOW IF S0 = 1 (ZAMBIA) AND LIST 1 = 1 (LUSAKA)**
33	KAFUE	
34	CHONGWE	
35	LUANGWA	
36	SOLWEZI	**SHOW IF S0 = 1 (ZAMBIA) AND LIST 1 = 1 (NORTH WESTERN)**
37	MWINILUNGA	
38	KABOMPO	
39	ZAMBEZI	
40	KASEMPA	
41	MUFUMBWE	
42	CHAVUMA	
45	MPIKA	**SHOW IF S0 = 1 (ZAMBIA) AND LIST 1 = 1 (MUCHINGA)**
46	ISOKA	
47	CHINSALI	
50	NAKONDE	
43	KASAMA	**SHOW IF S0 = 1 (ZAMBIA) AND LIST 1 = 1 (NORTHERN)**
44	MBALA	
48	MUNGWI	
49	LUWINGU	
51	KAPUTA	
52	MPOROKOSO	
53	MPULUNGU	
54	CHILUBI	
55	MAZABUKA	**SHOW IF S0 = 1 (ZAMBIA) AND LIST 1 = 1 (SOUTHERN)**
56	KALOMO	
57	CHOMA	
58	MONZE	
59	LIVINGSTONE	
60	SINAZONGWE	
61	NAMWALA	
62	KAZUNGULA	
63	SIAVONGA	
64	ITEZHI-TEZHI	

65	GWEMBE	
66	KAOMA	SHOW IF S0 = 1 (ZAMBIA) AND LIST 1 = 1 (WESTERN)
67	MONGO	
68	KALABO	
69	SENANGA	
70	SESHEKE	
71	SHANGAMBO	
72	LUKULU	
73	BULAWAYO	AUTOMATICALLY CODE IF S0 = 2 (ZIMBABWE) AND LIST 1 = 11 (BULAWAYO)
74	CHITUNGWIZA	AUTOMATICALLY CODE IF S0 = 2 (ZIMBABWE) AND LIST 1 = 12 (CHITUNGWIZA)
75	HARARE	AUTOMATICALLY CODE IF S0 = 2 (ZIMBABWE) AND LIST 1 = 13 (HARARE)
76	MUTARE	SHOW IF S0 = 2 (ZIMBABWE) AND LIST 1 = 14 (MANICALAND)
77	CHIPINGE	
78	MAKONI	
79	BUHERA	
80	MUTASA	
81	NYANGA	
82	CHIMANIMANI	
83	MAZOWE	SHOW IF S0 = 2 (ZIMBABWE) AND LIST 1 = 15 (MASHONALAND CENTRAL)
84	MT. DARWIN	
85	GURUVE	
86	BINDURA	
87	CENTENARY	
88	SHAMVA	
89	RUSHINGA	
90	GOROMONZI	SHOW IF S0 = 2 (ZIMBABWE) AND LIST 1 = 16 (MASHONALAND EAST)
91	SEKE	
92	MARONDERA	
93	MUREWA	
94	MUTOKO	
95	CHIKOMBA	
96	MUDZI	
97	UMP	
98	WEDZA	
99	HURUNGWE	SHOW IF S0 = 2 (ZIMBABWE) AND LIST 1 = 17 (MASHONALAND WEST)
100	KADOMA	
101	CHEGUTU	
102	ZVIMBA	
103	MAKONDE	

104	KARIBA	
105	MASVINGO	**SHOW IF S0 = 2 (ZIMBABWE) AND LIST 1 = 18 (MASVINGO)**
106	CHIREDZI	
107	GUTU	
108	ZAKA	
109	BIKITA	
110	CHIVI	
111	MWENEZI	
112	HWANGE	**SHOW IF S0 = 2 (ZIMBABWE) AND LIST 1 = 19 (MATABELALAND NORTH)**
113	TSHOLOTSHO	
114	BINGA	
115	NKAYI	
116	LUPANE	
117	UMGUZA	
118	BUBI	
119	GWANDA	**SHOW IF S0 = 2 (ZIMBABWE) AND LIST 1 = 20 (MATABELELAND SOUTH)**
120	BEIT BRIDGE	
121	BULILIMA	
122	MATOBO	
123	INSIZA	
124	MANGWE	
125	UMZINGWANE	
126	KWEKWE	**SHOW IF S0 = 2 (ZIMBABWE) AND LIST 1 = 21 (MIDLANDS)**
127	GOKWE SOUTH	
128	GWERU	
129	GOKWE NORTH	
130	MBERENGWA	
131	ZVISHAVANE	
132	SHURUGWI	
133	CHIRUMHANZU	
